# Systems Biology and Experimental Validation Enable Discovery of MMP9-Centered Networks, Anticancer Activity, and Pharmacodynamic Signature in Non-Small Cell Lung Cancer

**DOI:** 10.3390/ijms27146457

**Published:** 2026-07-20

**Authors:** Zainab Ahmed Rashid, Rima Hajjo, Dima A. Sabbah, Kamal Sweidan, Shriefa Almutairi, Sanaa K. Bardaweel

**Affiliations:** 1 Department of Pharmaceutical Sciences, School of Pharmacy, The University of Jordan, Amman 11942, Jordan; zainab.rashid1995@gmail.com (Z.A.R.); shriefaalmutairi@gmail.com (S.A.); 2 Department of Pharmacy, Faculty of Pharmacy, Al-Zaytoonah University of Jordan, P.O. Box 130, Amman 11733, Jordan; dima.sabbah@zuj.edu.jo; 3 Laboratory for Molecular Modeling, Division of Chemical Biology and Medicinal Chemistry, Eshelman School of Pharmacy, The University of North Carolina at Chapel Hill, Chapel Hill, NC 27599, USA; 4 Department of Chemistry, The University of Jordan, Amman 11942, Jordan; k.sweidan@ju.edu.jo

**Keywords:** matrix metalloproteinase-9 (MMP9) inhibitors, non-small cell lung cancer (NSCLC), systems biology, pharmacodynamic biomarker, extracellular matrix remodeling, apoptosis

## Abstract

Matrix metalloproteinase-9 (MMP9) is involved in extracellular matrix remodeling, inflammation, and metastasis, and its overexpression is associated with poor prognosis in lung cancer. However, the systems-level effects of MMP9 inhibition remain incompletely understood. We combined systems biology with experimental validation to characterize MMP9-centered signaling in non-small cell lung cancer (NSCLC) and identify downstream pharmacodynamic biomarkers. Network and pathway analyses revealed extracellular matrix- and inflammation-related interaction modules and prioritized an *MMP9*-associated gene panel. Synthesized compounds were evaluated using purified enzyme assays and A549 NSCLC cells. At 50 μM, compounds M34 and M33 showed the strongest inhibition of MMP9 activity (65.01% and 61.32%, respectively). Seven compounds (M1, M2, M8, M9, M10, M27, and M34) demonstrated antiproliferative activity at 72 h (IC_50_ = 40–115 μM), suppressed migration and colony formation, induced apoptosis, and reduced MMP9 protein expression. Quantitative PCR confirmed coordinated downregulation of *MMP9*, *VEGFA*, *APP*, and *ETV4*, with upregulation of *CDH1*, *COL5A1*, *COL6A2*, *CCL2*, and *CCL17*. Enrichment analysis linked these changes to inflammatory signaling, immune activation, and extracellular matrix remodeling. These findings establish an experimentally supported eight-gene pharmacodynamic biomarker signature and support biomarker-guided development of MMP9-targeted strategies in NSCLC.

## 1. Introduction

Matrix metalloproteinase-9 (MMP9; gelatinase B) is a zinc-dependent endopeptidase that participates in extracellular matrix (ECM) remodeling and thereby facilitates key steps in tumor progression, including invasion and metastasis [1,2]. MMP9 expression is regulated at multiple levels, including epigenetic control and non-coding RNA–mediated mechanisms, which collectively shape its cell- and context-specific activity [1,3]. In addition, inflammatory mediators can amplify MMP9 expression through coordinated signaling and transcriptional programs, including NF-κB-dependent regulation, linking MMP9 activity to tumor microenvironment crosstalk [4,5]. Given these regulatory layers and the dynamic nature of ECM remodeling, MMP9 is increasingly recognized as a mechanistically important node in cancer biology and a promising target for inhibitor development [6].

In lung cancer, multiple lines of evidence support MMP9 as a clinically relevant axis. Promoter polymorphisms have been associated with altered MMP9 expression and clinical outcome in non-small cell lung cancer (NSCLC), indicating that inherited variation can influence MMP9-driven disease behavior [7]. Consistent with this, meta-analytic evidence indicates that elevated MMP9 in serum and/or tissue correlates with poorer prognosis in NSCLC, reinforcing its biomarker relevance [8]. At the same time, while MMP9 has been proposed as a target and biomarker and biosensing strategies continue to advance, the systems-level consequences of MMP9 inhibition remain insufficiently defined in lung cancer settings [9,10,11,12]. Specifically, it is still unclear which downstream programs are coherently modulated and which readouts best report pathway engagement [10].

Network and pathway interrogation approaches can help bridge this gap by integrating curated molecular interactions to reconstruct mechanistic modules surrounding a target and nominate measurable downstream readouts that can serve as pharmacodynamic (PD) biomarkers. Modern representation learning and network analysis frameworks further support systematic inference over complex biological graphs, improving the interpretability of target-centered mechanisms [13,14]. Previous studies, including our own, have applied integrative systems biology workflows to connect computational inference with experimental validation, emphasizing mechanism-anchored marker nomination and testing [15,16,17].

Here, we present an integrated systems biology and experimental strategy to elucidate the mechanisms of MMP9 inhibition and define a pharmacodynamic (PD) biomarker signature in NSCLC. Specifically, we reconstructed MMP9-centered interaction networks and functional modules using curated knowledge bases, network visualization platforms, and pathway inference approaches. These analyses prioritized a downstream PD gene signature representing key MMP9-regulated processes, which was subsequently evaluated alongside a panel of synthesized MMP9 inhibitors in A549 NSCLC cells to validate phenotypic and molecular responses. By linking mechanistic network analysis with an experimentally supported PD signature, this study provides a practical framework for biomarker-informed assessment of MMP9 pathway modulation in lung cancer.

## 2. Results

### 2.1. Systems Biology Analysis Reveals MMP9 as a Central Regulator of Extracellular Matrix and Inflammatory Signaling in Lung Cancer

**Figure 1:**
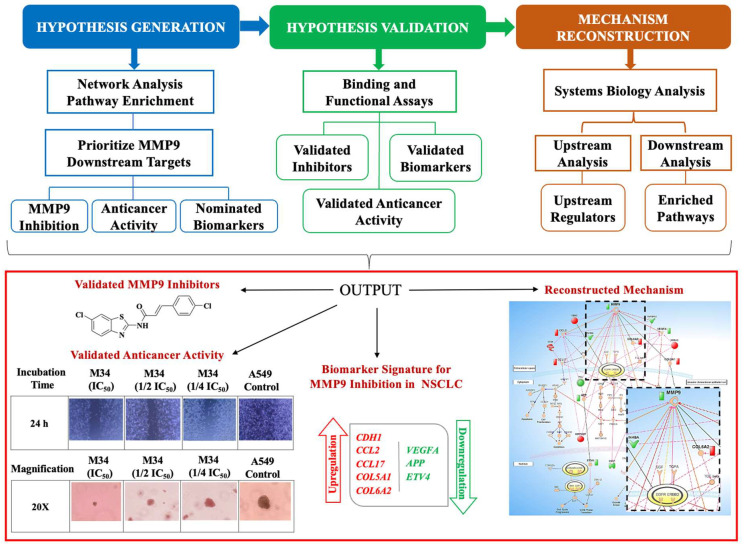
Integrative workflow linking bioinformatics, experimental validation, and mechanistic reconstruction of MMP9 in NSCLC.

**Figure 2:**
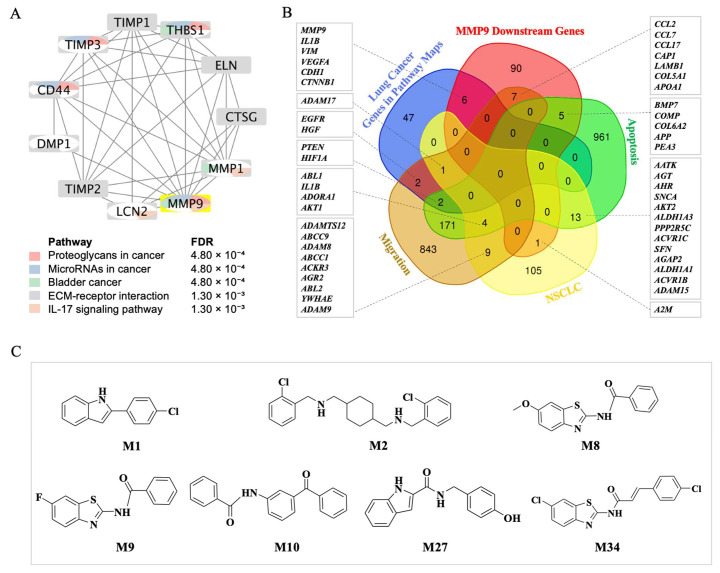
Network-based systems biology analysis of MMP9 in NSCLC and chemical structures of synthesized inhibitors. (**A**) Protein–protein interaction (PPI) network of MMP9 showing nearest-neighbor interactions with extracellular matrix components, adhesion molecules, protease inhibitors, and inflammatory mediators. The yellow box represents the seed node. (**B**) Gene-set overlap analysis linking MMP9 downstream targets to lung cancer-related pathways and functional gene sets, including apoptosis, migration, and NSCLC. (**C**) Chemical structures of the synthesized MMP9 inhibitors evaluated in the present study.

A systems biology analysis integrating curated pathway maps, protein–protein interaction (PPI) context, and functional enrichment was performed to define the network role of MMP9 in lung cancer (Figure 1). Nearest-neighbor PPI analysis identified a highly connected MMP9–centered module enriched for ECM components, endogenous metalloproteinase inhibitors, adhesion molecules, and inflammatory mediators (Figure 2A). Prominent interactors included matrix proteases (e.g., MMP1), tissue inhibitors (TIMP1–3), ECM structural proteins (e.g., ELN, THBS1), and adhesion regulators (e.g., CD44), consistent with a tumor-microenvironment remodeling function. Pathway enrichment of the PPI neighborhood showed significant overrepresentation of cancer- and inflammation-relevant pathways, including ECM–receptor interaction, IL-17 signaling, and proteoglycans in cancer, supporting a multifunctional role for MMP9 at the interface of matrix turnover, immune signaling, and oncogenic programs. Collectively, these results nominate MMP9 as a central network hub linking structural remodeling with inflammatory cues in lung cancer (Figure 2A). Having established the central network position of MMP9, we next investigated its involvement within lung cancer-specific signaling pathways to identify the molecular programs and downstream effectors most closely associated with its regulatory function.

### 2.2. Integration of Lung Cancer Pathway Maps and Cancer-Associated Gene Sets Identifies Core MMP9–Linked Signaling Modules

To contextualize *MMP9* within lung cancer–specific signaling programs, MetaCore^TM^ pathway mining identified seven lung cancer canonical pathway maps containing *MMP9*, spanning *EGFR*, *IGF*, *KRAS*, *HGF/MET*, *WNT* inhibition, smoking-associated *EGFR* activation, and *TGF-β* signaling. Gene content across these maps was compiled to identify recurrent network objects shared across pathways; the overlap summary is provided as Supporting Information Table S2. In parallel, cancer-associated gene sets were assembled using MetaCore™ advanced searches for apoptosis (1157 genes), migration (1040 genes), and NSCLC (134 genes).

Integration of (i) genes present on the seven *MMP9*–containing lung cancer pathway maps, (ii) curated direct downstream targets of *MMP9*, and (iii) apoptosis/migration/NSCLC gene sets revealed a focused subset of overlapping genes that recurrently link *MMP9* to lung-cancer processes (Figure 2B). Overlaps highlighted inflammatory mediators (*IL1B*, *CCL2*, *CCL17*), EMT/adhesion regulators (*CDH1*, *VIM*), angiogenic signaling (*VEGFA*), and ECM structural genes (*COL5A1*, *COL6A2*), providing a rational pool for downstream prioritization. Curated outgoing interaction mining further identified 83 experimentally validated direct downstream targets of *MMP9* in MetaCore™, partitioned into 17 activated and 66 inhibited targets (Table 1). Activated targets included latent signaling molecules and cytokines/growth factors (e.g., *TGFB1/2/3*, *VEGFA*, *IL1B*, *CXCL8*) and the transcription factor *ETV4*, whereas inhibited targets were enriched for ECM/adhesion/tight-junction components (e.g., multiple collagens including *COL5A1/COL6A2*, *ELN*, *FN1*, *LAMB1*, *CDH1*, *CLDN5*) and immune chemokines (e.g., *CCL2*, *CCL17*). These data support the dual functional logic of *MMP9* in lung cancer: activation of latent signaling programs alongside proteolytic degradation of structural barriers. To prioritize the most biologically relevant downstream effectors for experimental validation, these integrated datasets were subsequently subjected to a systematic multi-parameter scoring strategy.

### 2.3. Multi-Parameter Scoring Prioritizes an Eight-Gene MMP9–Associated Candidate Pharmacodynamic Signature

Genes emerging from overlap analyses were prioritized using a structured multi-parameter scoring system (score range: 0–6) incorporating: (i) direct interaction with MMP9, (ii) recurrence across lung cancer pathway maps, (iii) apoptosis association, (iv) migration association, (v) NSCLC relevance, and (vi) prior experimental evidence/assay feasibility. This framework nominated eight high-confidence genes (score ≥ 4) as a focused *MMP9*–associated panel: *VEGFA*, *CDH1*, *CCL2*, *CCL17*, *COL6A2*, *COL5A1*, *APP*, and *ETV4* (Table 2). Seven genes achieved the maximum observed score (5/6: *VEGFA*, *CDH1*, *CCL2*, *CCL17*, *COL6A2*, *COL5A1*, *APP*), while *ETV4* scored 4/6 and was retained because it represented the only transcriptional regulator in the prioritized set with direct MMP9 linkage in MetaCore™.

Based on curated directionality annotations, *VEGFA*, *ETV4*, and *APP* were predicted to be activated by *MMP9* and therefore expected to decrease upon inhibition, whereas *CDH1*, *CCL2*, *CCL17*, *COL5A1*, and *COL6A2* were predicted to be inhibited by *MMP9* and therefore expected to increase upon inhibition. This scoring strategy yielded a concise, mechanistically interpretable gene panel suitable for downstream transcriptional/protein-level validation and pharmacodynamic monitoring. Before proceeding to experimental validation, we further examined whether the prioritized genes represented coherent biological processes and signaling pathways relevant to NSCLC progression.

### 2.4. Systems Biology Context of the Prioritized Gene Signature

To place the eight-gene panel into a functional systems context, canonical pathway enrichment analysis was performed in IPA. Enrichment results organized the signature into two interconnected biological programs central to lung-cancer progression. The first program reflected inflammatory/immune activation, with strong enrichment in pathways such as Pathogen-Induced Cytokine Storm Signaling and *IL-17* Signaling, consistent with cytokine regulation and immune recruitment via *CCL2/CCL17* and inflammatory coupling to *MMP9* biology. The second program reflected ECM remodeling and tissue reorganization, supported by enrichment in Wound Healing Signaling, Assembly of collagen fibrils, and Collagen degradation, consistent with coordinated regulation of *COL5A1/COL6A2* and matrix turnover downstream of MMP9 activity.

The top enriched canonical pathways and their associated false discovery rates are summarized in Table 3. These analyses indicate that *VEGFA*, *CDH1*, *CCL2*, *CCL17*, *COL5A1*, *COL6A2*, *APP*, and *ETV4* form a coherent regulatory module linking inflammation, angiogenesis, epithelial–mesenchymal plasticity, and ECM remodeling, providing mechanistic support for this panel as a pharmacodynamic readout of *MMP9* pathway engagement. These computational findings provided the rationale for experimental validation to determine whether inhibition of MMP9 produces the predicted molecular and phenotypic responses in NSCLC cells.

### 2.5. Experimental Biological Evaluation Supports Functional and Molecular Consequences of MMP9 Inhibition

To validate the systems biology predictions, we implemented a multilayer experimental cascade spanning pure enzyme inhibition, cellular phenotypes, and molecular target engagement. MMP9 enzymatic inhibition was determined for newly synthesized inhibitors M33–M41, while inhibition data for M5–M15 and M20–M29 were reported previously [34]. Synthetic procedures and structure elucidation for M33–M41 are provided in the Supplementary Material (Supplementary Figures S1–S6; Schemes S1 and S2). Functional cellular activity was assessed via antiproliferative testing in NSCLC lines (A549, H1299, H661) over 48–96 h (Supplementary Table S3), followed by phenotypic assays of migration (wound healing at IC_50_, ½IC_50_, ¼IC_50_; Supplementary Figure S7 and Table S4) and anchorage-independent growth (soft agar; Supplementary Figure S8 and Table S5). Molecular target engagement was examined by qRT-PCR (*MMP9* and prioritized downstream targets at 0.1 × IC_50_; Supplementary Figure S9) and by Western blotting (MMP9 protein at ¼IC_50_ and ½IC_50_). Finally, to determine the cellular mechanisms underlying the observed biological effects, flow cytometry was performed to evaluate alterations in cell-cycle progression and apoptosis (Supplementary Figures S11–S14). Together, these complementary assays establish a sequential experimental validation pipeline linking MMP9 inhibition with phenotypic and molecular responses in NSCLC cells.

#### 2.5.1. MMP9 Inhibitors Reduce Purified Enzyme Activity

Purified MMP9 inhibition by the synthesized cinnamamides was assessed using a colorimetric assay [34]. At 50 µM, M34 showed the highest inhibitory activity (65.01%), followed by M33 (61.32%). Additional compounds showed low-to-moderate inhibition, including M41 (42.91%), M39 (22.65%), M35 (22.28%), and M40 (20.81%), indicating variable enzyme-level potency across the series. These enzyme inhibition results provided the initial evidence of target engagement and justified subsequent evaluation of the compounds in cellular models to determine whether biochemical inhibition translated into anticancer activity.

#### 2.5.2. MMP9 Inhibitors Suppress NSCLC Cell Viability with Time-Dependent Potency

**Figure 3:**
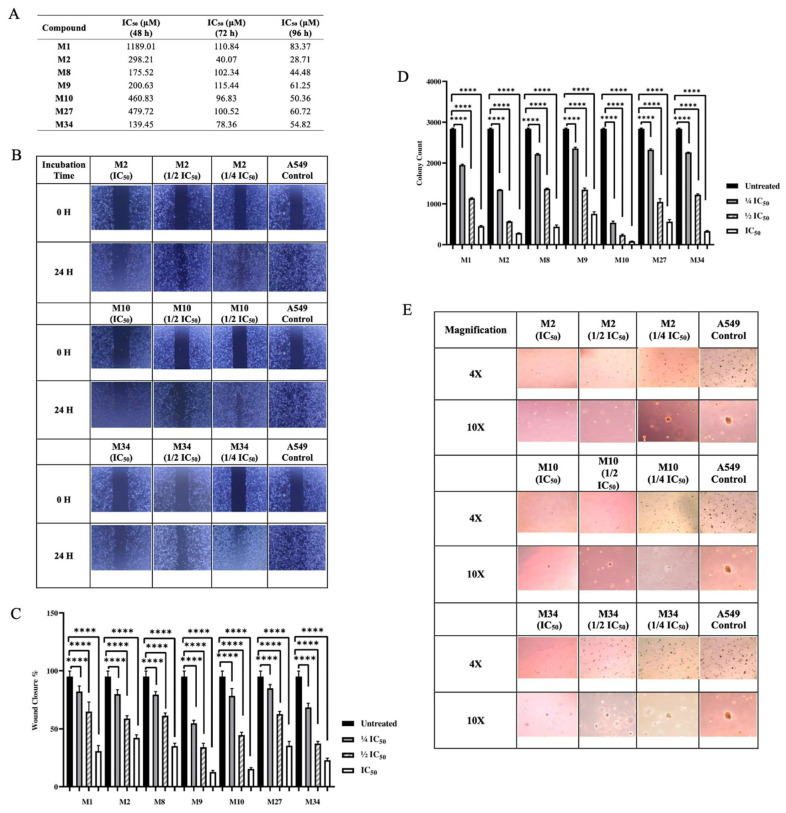
MMP9 inhibitors suppress cell viability, migration and clonogenicity in A549 cells. (**A**) IC_50_ (µM) values for MMP9 inhibitors in A549 cells at 48, 72, and 96 h (SD never exceeded 8%; experiments run in duplicate and repeated three independent times, *n* = 6). (**B**) Wound closure after 24 h treatment at concentrations relative to IC_50_ (microscope magnification 10×) (**C**) Quantitative analysis of wound closure, showing the percentage of wound closure relative to the untreated area. (**D**) Quantitative analysis of colony formation, showing the relative colony number normalized to the untreated control. (**E**) Soft-agar colony formation following 72 h pretreatment (microscope magnification 4× and 10×). Data represent mean ± SD from *n* independent experiments normalized to untreated controls. Statistical significance was determined using GraphPad’s Prism 9 (**** *p* ≤ 0.0001).

Antiproliferative effects were evaluated by MTT assay in A549, H1299, and H661 cells after exposure to increasing compound concentrations for 48, 72, and 96 h. Across the dataset, compounds reduced viability in a dose-dependent manner relative to controls (Supplementary Table S3). The most active compounds were M1, M2, M8, M9, M10, M27, and M34 (Figure 3A). In A549 cells at 72 h, IC_50_ values were 110.84 µM (M1), 40.07 µM (M2), 102.34 µM (M8), 115.44 µM (M9), 96.83 µM (M10), 100.52 µM (M27), and 78.36 µM (M34). These time-dependent IC_50_ values in A549 (Table 4) guided selection of M1, M2, M8, M9, M10, M27, and M34 for subsequent phenotypic and mechanistic assays. Furthermore, normal dermal fibroblasts showed good tolerability at the tested concentrations. Based on their favorable antiproliferative activity, M1, M2, M8, M9, M10, M27, and M34 were selected for subsequent phenotypic and mechanistic studies to determine whether reduced cell viability was accompanied by suppression of other malignant characteristics of NSCLC cells.

#### 2.5.3. MMP9 Inhibitors Impair A549 Migration and Anchorage-Independent Growth

In wound-healing assays, A549 cells treated with M1, M2, M8, M9, M10, M27, and M34 at IC_50_, ½IC_50_, and ¼IC_50_ showed significant, concentration-dependent inhibition of migration compared with untreated cells (Figure 3B,C; Supplementary Figure S7 and Table S4). At IC_50_, migration inhibition was approximately 77% (M34), 66% (M8), 87% (M9), 83% (M10), 61% (M27), 71% (M1), and 54% (M2) (*p* < 0.0001). Anchorage-independent growth was evaluated by soft agar following 72 h pretreatment with compounds at IC_50_, ½IC_50_, and ¼IC_50_. Relative to untreated cells, all tested inhibitors significantly reduced colony number and size (Figure 3D,E; Supplementary Figure S8 and Table S5). These findings demonstrate that MMP9 inhibition suppresses not only cell proliferation but also migratory and clonogenic capacities. We next investigated whether these phenotypic effects were associated with the transcriptional changes predicted by the systems biology analyses.

#### 2.5.4. qRT-PCR Confirms MMP9 Suppression and Directionally Coherent Modulation of the Prioritized Gene Panel

To test whether MMP9 inhibition produces the transcriptional shifts predicted by the systems biology model, qRT-PCR was performed in A549 cells following treatment with M1, M2, M8, M9, M10, M27, and M34 at 0.1 × IC_50_. Baseline profiling showed *MMP9* to be overexpressed in A549 relative to H1299 and H661 (Figure S9). Relative to untreated cells, all seven compounds significantly downregulated *MMP9* mRNA in A549 (*p* < 0.0001) (Table 4).

Across downstream targets, *VEGFA*, *APP*, and *ETV4* were significantly downregulated, whereas *CDH1*, *CCL2*, *CCL17*, *COL6A2*, and *COL5A1* were significantly upregulated (*p* < 0.0001) (Table 4). This directionality is broadly consistent with the curated interaction annotations used in the prioritization framework, with *APP* representing the main discordant component at the validation stage (addressed in Section 2.6. The observed transcriptional responses suggested effective modulation of the predicted MMP9 regulatory network. To determine whether these molecular changes extended to the protein level, MMP9 expression was subsequently examined by Western blot analysis.

#### 2.5.5. Western Blotting Supports Reduced MMP9 Protein Expression in A549 Cells

**Figure 4:**
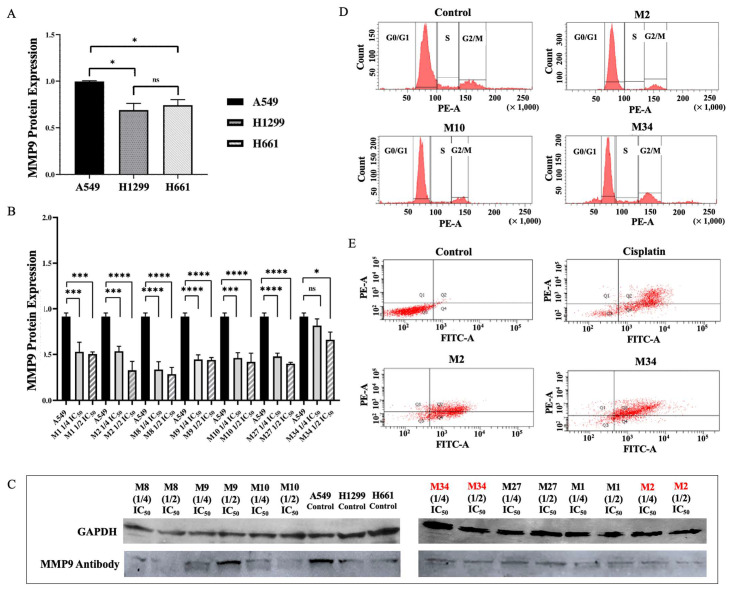
Effects of MMP9 inhibition on MMP9 protein expression in lung cancer cell lines. (**A**) Normalized basal MMP9 protein expression in untreated A549, H1299, and H661 lung cancer cell lines. (**B**) Quantitative analysis of MMP9 protein levels in A549 cells treated with MMP9 inhibitors at ¼IC_50_ and ½IC_50_ concentrations, normalized to GAPDH. Statistical significance was determined using GraphPad’s Prism 9 (* *p* ≤ 0.01, *** *p* ≤ 0.001, **** *p* ≤ 0.0001). ns: not significant. (**C**) Representative cropped Western blot showing MMP9 protein expression in A549 cells following treatment with MMP9 inhibitors compared to untreated control. Compounds highlighted in red (M34 and M2) represent the most potent MMP9 inhibitors based on IC_50_ values. Protein expression was evaluated by densitometric analysis of immunoblot bands. MMP9, matrix metalloproteinase-9; GAPDH, glyceraldehyde 3-phosphate dehydrogenase. Original blots are presented in the Supplementary Figure S10. (**D**) Effect of MMP9 inhibitors for 48 h using ½IC_50_ treatment on the cell cycle of the A549 cell line. Histogram of DNA content upon PI staining of respective samples showing G0/G1, S, and G2 phases of the cell cycle. μM: micromolar. (**E**) Apoptosis analysis by Annexin V-FITC/PI dual staining in A549 cells. Representative dot plots show the distribution of cells across four populations: viable (Annexin^−^/PI^−^, **lower left**), early apoptotic (Annexin^+^/PI^−^, **lower right**), late apoptotic (Annexin^+^/PI^+^, **upper right**), and necrotic (Annexin^−^/PI^+^, **upper left**).

Western blot analysis showed that basal MMP9 protein expression was higher in A549 compared with H1299 and H661 (Figure 4A). In A549 cells, treatment with M1, M2, M8, M9, M10, M27, and M34 at ¼IC_50_ and ½IC_50_ resulted in a significant reduction in MMP9 protein expression relative to untreated controls (*p* < 0.0001) (Figure 4B,C). The reduction in MMP9 protein expression confirmed target modulation at both the transcriptional and protein levels. We therefore proceeded to investigate the cellular mechanisms responsible for the observed antitumor effects by examining cell-cycle progression and apoptosis.

#### 2.5.6. MMP9 Inhibitors Alter Cell-Cycle Distribution and Induce Apoptosis in A549 Cells

To determine the mechanisms underlying the observed reductions in proliferation, migration, and clonogenic growth, we evaluated the effects of MMP9 inhibition on cell-cycle progression and apoptosis in A549 cells. Cell-cycle analysis by PI staining after 48 h treatment at ½IC_50_ showed that M1, M2, M10, and M27 induced a significant G0/G1 arrest, whereas M8 and M34 produced a pronounced G2/M arrest (Figure 4D, Supplementary Figures S11 and S12).

Apoptosis analysis by Annexin V-FITC/PI staining demonstrated a marked increase in early and late apoptotic populations (Q2 + Q4) in A549 cells treated with MMP9 inhibitors (Figure 4E). Total apoptosis increased to 80.9% (M1), 86.9% (M2), 79.4% (M8), 74.4% (M9), 72.5% (M10), 71.8% (M27), and 87.2% (M34) compared with 2.2% in untreated cells. Cisplatin produced 88.8% apoptosis and was comparable to M1, M2, and M34, while exceeding M8, M9, M10, and M27. Quantitative phase comparisons are provided in Supplementary Figures S13 and S14. Having demonstrated that MMP9 inhibition induces consistent molecular and phenotypic alterations, we next assessed whether the experimentally observed gene expression changes supported the computationally predicted pharmacodynamic signature.

### 2.6. Experimental Validation Supports a Pharmacodynamic Gene Panel for MMP9 Inhibition in NSCLC

To validate the computational predictions at the biomarker-signature level, qRT-PCR results were compared with MetaCore™ directionality annotations for the prioritized panel. Seven genes (*VEGFA*, *CDH1*, *CCL2*, *ETV4* (*PEA3*), *COL6A2*, *CCL17*, *COL5A1*) exhibited expression changes consistent with predicted directionality following MMP9 inhibition (Table 5), supporting their placement within an MMP9–regulated network module. *APP* was discordant, showing downregulation rather than the predicted increase, suggesting context-dependent regulation or indirect coupling under these experimental conditions.

To assess translational relevance, IPA Biomarker Filter annotation indicated that *CCL2*, *ETV4*, *CDH1*, *MMP9*, and *VEGFA* are established lung-cancer biomarkers, whereas *CCL17*, *APP*, *COL5A1*, and *COL6A2* are not currently annotated, indicating potential novelty as pharmacodynamic readouts of MMP9 modulation. Together, these data support an eight-gene pharmacodynamic panel for monitoring MMP9–targeted interventions in NSCLC: *VEGFA*, *CDH1*, *CCL2*, *ETV4*, *COL6A2*, *CCL17*, *COL5A1*, and *APP* (Table 5). To further place these experimentally validated biomarkers into a broader biological context, we reconstructed the underlying regulatory network and identified the upstream signaling mechanisms associated with the observed transcriptional responses.

### 2.7. Mechanistic Reconstruction Implicates PKC-Centered Signaling, EMT Suppression, and Coordinated Remodeling Programs Downstream of MMP9 Inhibition

#### 2.7.1. IPA Causal Network Analysis Identifies Upstream Regulators Associated with Transcriptional Responses to MMP9 Inhibition

IPA Causal Network Analysis was used to infer upstream regulators driving the observed transcriptional response to compounds M1, M2, M8, M9, M10, M27, and M34. The inferred architecture implicated inhibited kinase signaling, altered non-coding RNA activity, and suppression of pro-metastatic transcriptional programs (Table 5). PKC emerged as the most significant upstream regulator (*p* = 9.95 × 10^−11^) and was predicted to be inhibited (Z = −1.000), consistent with coordinated decreases in MMP9, VEGFA, and CCL2 and attenuation of invasion/angiogenesis/inflammatory signaling. NORAD showed predicted activation (Z = 2.000), whereas TERC was predicted to be inhibited (Z = −1.732). The EMT-associated transcription factor SNAI1 was predicted to be inhibited (Z = −0.577), aligning with CDH1 upregulation and a less invasive phenotype. Additional regulators (INHBA, FBN2, TNFSF12) were predicted to be inhibited, while SERPINB7 showed mild predicted activation (Z = 0.577), potentially reflecting compensatory rebalancing of proteolysis. The predicted upstream regulators were subsequently integrated with the validated downstream targets to reconstruct the signaling network underlying the biological effects of MMP9 inhibition.

#### 2.7.2. Network Reconstruction Integrates Validated Targets and Upstream Regulators into a Coordinated Anti-Tumor Mechanism

**Figure 5:**
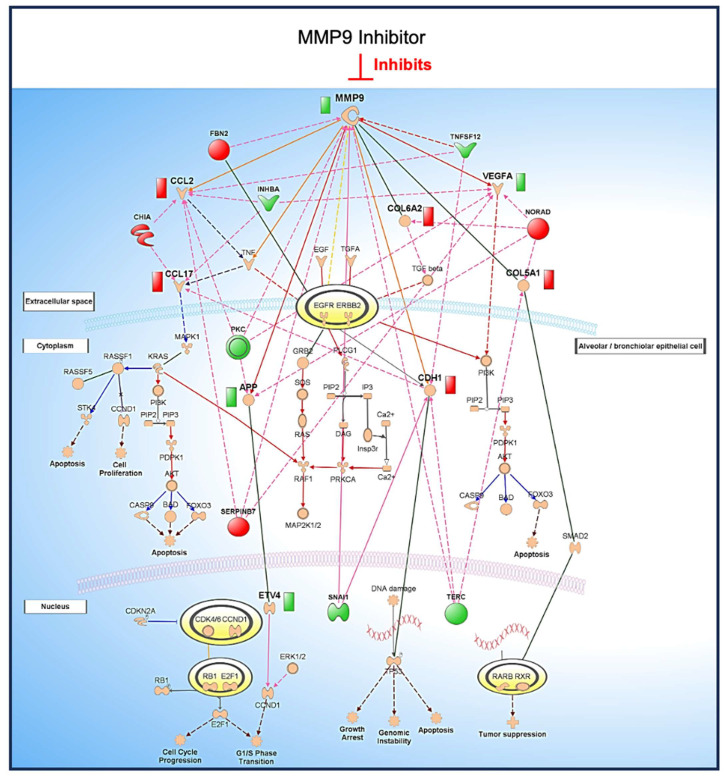
Lead MMP9 inhibitors and reconstructed signaling network in NSCLC. The top panel shows the chemical structures of the seven validated MMP9 inhibitors (M1, M2, M8, M9, M10, M27, M34). The bottom panel presents the reconstructed mechanistic network integrating upstream master regulators (predicted via IPA Causal Analysis) and downstream effectors validated by qRT-PCR following treatment with MMP9 inhibitors. Yellow nodes represent receptor complexes, whereas orange nodes denote different classes of molecules, including transcription factors, cytokines, growth factors, kinases, and peptides.Node colors indicate expression changes after inhibition: red = upregulated, green = downregulated. Edge styles and colors denote interaction types: solid lines = direct interactions; dashed lines = indirect interactions; red edges = activation; blue edges = inhibition; green edges = protein–protein interaction, orange edges = molecular cleavage, purple edges = localization, grey edges = reactions, dark red edges = causation, dark yellow edges = phosphorylation, yellow edges = transcription, and pink edges = expression.Cellular compartments provide spatial context for network localization.

A mechanistic network was reconstructed using the validated downstream genes (*VEGFA*, *CCL2*, *CCL17*, *COL5A1*, *COL6A2*, *APP*, *ETV4*, *CDH1*) and the inferred upstream regulators. The reconstructed map positioned MMP9 as a central extracellular hub connecting inflammatory signaling, ECM remodeling, proliferative pathways, and angiogenesis (Figure 5). Transcriptional profiling after inhibition showed coherent changes across this network: *VEGFA*, *APP*, *CCL2*, *ETV4*, and *MMP9* were downregulated, whereas *CDH1*, *CCL17*, *COL5A1*, and *COL6A2* were upregulated, linking target engagement to phenotypic outcomes in migration, clonogenicity, cell-cycle distribution, and apoptosis (Section 2.5.3, Section 2.5.4, Section 2.5.5 and Section 2.5.6). Finally, the combined computational and experimental findings were consolidated into a pharmacodynamically relevant biomarker panel for monitoring MMP9 pathway modulation in NSCLC.

#### 2.7.3. Pharmacodynamic Biomarker Signature Nomination Supported by Directional Experimental Validation

Based on the integrative systems biology framework, we propose a focused pharmacodynamic (PD) biomarker panel reflecting key MMP9–regulated processes in NSCLC: angiogenesis (*VEGFA*), EMT/adhesion (CDH1, ETV4), immune recruitment/inflammation (*CCL2*, *CCL17*), and ECM remodeling (*COL5A1*, *COL6A2*), with *APP* retained as a context-dependent component. In A549 cells, treatment with M1, M2, M8, M9, M10, M27, and M34 produced directionally consistent transcriptional responses across the panel, supporting its use as a mechanistically anchored PD readout of MMP9 pathway modulation rather than a single-compound signature (Table 5). The consolidated directional behavior, biological interpretation, and proposed clinical readouts of this pharmacodynamic biomarker panel are summarized in Table 5. Importantly, this panel is compatible with clinically feasible measurement approaches, including qRT-PCR from tumor tissue and multiplex protein quantification for secreted mediators (e.g., *CCL2*, *CCL17*, *VEGFA*). This PD signature is proposed for monitoring target engagement and pathway modulation during MMP9–directed interventions, not as a diagnostic classifier.

## 3. Discussion

This study demonstrates an integrative systems biology and experimental pharmacology framework to translate MMP9 from a broad protease target into a mechanistically anchored therapeutic hypothesis in NSCLC. By moving beyond empirical screening, we utilized curated network analysis to map MMP9 as a systems-level hub within critical oncogenic pathways, including *EGFR/MAPK* and *PI3K/AKT* signaling. Mining lung cancer pathway maps, direct downstream interactions, and cancer-associated gene sets (apoptosis, migration, NSCLC) allowed us to define the pathways and phenotypes most likely influenced by MMP9 activity. Consequently, experimental readouts were selected a priori to test these predictions, encompassing MMP9 enzymatic inhibition, proliferation, migration, clonogenicity, cell-cycle distribution, apoptosis, and modulation of a prioritized downstream gene panel, thereby establishing a direct bridge between computational prediction and biological validation.

Our network analysis confirmed that MMP9 functions not merely as an ECM-degrading protease, but as a systems-level hub embedded within critical oncogenic pathways, including ECM–receptor interaction [35], *IL-17* signaling [36], *EGFR/MAPK*, *PI3K/AKT*, and *TGF-β* signaling [37,38]. The nearest-neighbor PPI network revealed its interplay with matrix components, adhesion molecules, and inflammatory mediators. The overlap analysis of lung cancer maps with apoptosis, migration, and NSCLC gene sets pinpointed key intersection nodes, including VEGFA, CDH1, CCL2, VIM, IL1B, and CTNNB1, suggesting that MMP9 inhibition would coordinately affect angiogenesis, epithelial–mesenchymal transition (EMT), inflammatory recruitment, and matrix remodeling.

Experimental validation strongly supported this informatics-driven prioritization. Biochemically, synthesized cinnamamide derivatives inhibited MMP9 enzymatic activity. Although M34 showed the greatest MMP9 enzymatic inhibition, it did not exhibit the lowest cellular IC_50_, whereas M1 demonstrated greater antiproliferative activity despite more moderate enzyme inhibition. This difference likely reflects the influence of cellular pharmacological factors (e.g., cell permeability, intracellular exposure, metabolic stability, protein binding, and engagement of downstream signaling pathways) beyond enzyme inhibition, underscoring the importance of combining biochemical and cell-based assays when evaluating MMP9 inhibitors. In NSCLC cell lines, the most active compounds (M1, M2, M8, M9, M10, M27, M34) reduced viability, impaired 2D migration, and suppressed clonogenic growth, with minimal toxicity toward normal fibroblasts, underscoring the role of MMP9 in the adhesive crosstalk that drives cancer cell motility [39]. These phenotypic effects were accompanied by robust downregulation of MMP9 mRNA and protein, cell-cycle arrest (G0/G1 or G2/M), and marked apoptosis induction comparable to cisplatin.

To translate network-level predictions into experimentally tractable readouts, we applied a transparent multi-criteria prioritization framework to derive a focused eight-gene pharmacodynamic signature of MMP9 inhibition. This approach integrated evidence of direct interaction with MMP9, recurrence across lung cancer pathway maps, association with apoptosis, migration, and NSCLC-related gene sets, and prior experimental support, resulting in prioritization of *VEGFA*, *CDH1*, *CCL2*, *CCL17*, *COL6A2*, *COL5A1*, *APP*, and *ETV4*. Pathway enrichment analysis revealed convergence on two tightly coupled biological programs, immune and inflammatory activation and extracellular matrix remodeling, which are central to NSCLC progression and directly actionable through MMP9 modulation [40]. Biomarker annotation using IPA indicated that *CCL2*, *CDH1*, *MMP9*, and *VEGFA* are established lung cancer biomarkers, whereas *COL5A1*, *COL6A2*, *CCL17*, and *APP* represent previously unannotated candidates, nominating them as novel pharmacodynamic readouts of MMP9 pathway blockade. Experimental validation by qPCR showed that seven of the eight prioritized genes exhibited directionally consistent transcriptional responses following inhibitor treatment, characterized by downregulation of pro-angiogenic and pro-invasive factors (*VEGFA*, *ETV4*) and upregulation of epithelial, immune, and matrix-associated genes (*CDH1*, *CCL2*, *CCL17*, *COL5A1*, *COL6A2*). The single discordant target, *APP*, likely reflects indirect or context-dependent regulation. Collectively, these findings validate the eight-gene panel as a mechanistically anchored pharmacodynamic signature reporting on angiogenesis, epithelial–mesenchymal plasticity, immune signaling, and extracellular matrix architecture downstream of MMP9 inhibition in NSCLC.

Mechanistically, IPA causal network analysis indicated that MMP9 inhibition is associated with predicted suppression of key upstream regulators, including PKC, SNAI1, TERC, INHBA, FBN2, and TNFSF12, alongside activation of NORAD and SERPINB7. These regulators link MMP9 activity to epithelial–mesenchymal transition (EMT) [41], telomerase-driven survival [42], *TGF-β*–mediated matrix remodeling [43], and apoptotic control [38]. Consistent with reports implicating MMP9 in *AKT* activation downstream of *EGFR* and *IGFR* signaling [38], protein kinases emerged as key inhibited regulators. Network reconstruction positioned MMP9 as a central extracellular amplifier coordinating angiogenesis (*VEGFA*), immune recruitment (*CCL2/CCL17*), extracellular matrix remodeling (*COL5A1/COL6A2*), and proliferative signaling via *EGFR*–*MAPK* and *PI3K/AKT* pathways. Its inhibition disrupts these parallel oncogenic programs, producing broad anti-tumor effects [44,45] beyond proteolysis. Notably, MMP9 blockade enhances apoptosis by preventing proteolytic cleavage of regulators such as E-cadherin; cleavage of mature E-cadherin generates soluble E-cadherin that can interact with *TP53* and attenuate its tumor-suppressive pro-apoptotic function [46]. Concurrent *CDH1* upregulation and *ETV4* downregulation reflect a coordinated shift toward epithelial stabilization and reduced invasiveness [47]. Collectively, these findings establish a unified mechanism linking MMP9 inhibition to coordinated multi-pathway suppression and the observed cytostatic and anti-migratory phenotypes in NSCLC.

Although the antiproliferative activity of the synthesized compounds was confirmed across three NSCLC cell lines (A549, H1299, and H661), detailed mechanistic studies were performed in A549 cells. This cell line is a well-established model for investigating MMP9-mediated signaling, extracellular matrix remodeling, and lung cancer progression [48,49], and it exhibited the highest basal MMP9 expression among the tested cell lines. Importantly, the proposed pharmacodynamic signature was not derived solely from A549 experiments but originated from integrative network, pathway, and target-prioritization analyses and was subsequently validated experimentally in A549 cells. Nevertheless, confirmation of the observed molecular responses and reproducibility of the eight-gene pharmacodynamic signature in additional NSCLC models representing distinct molecular subtypes would further strengthen confidence in its biological robustness and broader applicability. Future studies will therefore extend validation of the proposed biomarker panel to additional cellular and in vivo models.

In addition, although the synthesized compounds demonstrated inhibitory activity against MMP9, their selectivity for MMP9 relative to other MMP family members was not evaluated in the present study. Future investigations involving a broader panel of MMP isoforms will be important for establishing their selectivity profiles and further characterizing their pharmacological properties.

Our integrative approach, combining network biology, bioinformatics, and multi-level experimental validation, provides a robust framework for elucidating target mechanisms and nominating pharmacodynamic biomarkers. Using purified enzyme assays, cellular phenotyping, and molecular validation, we identify MMP9 as a therapeutically relevant node in NSCLC and validate an eight-gene signature as a pharmacodynamic readout of MMP9 inhibition. This panel captures angiogenic, inflammatory, epithelial, and extracellular matrix-related processes downstream of MMP9. Beyond characterizing a new class of MMP9 inhibitors, this study establishes a generalizable workflow for mechanism-informed biomarker discovery and translational monitoring of targeted therapies in oncology.

## 4. Materials and Methods

### 4.1. Computational Systems Biology and Network Analysis

#### 4.1.1. Databases and Software

MetaCore^TM^ version 21.4 (build 70,700) [50], Ingenuity Pathway Analysis (IPA) version 24.0 (QIAGEN) [51], and Cytoscape version 3.10.3 [52] were used as curated knowledge bases and network analysis platforms to reconstruct the molecular network of MMP9 in lung cancer. MetaCore™ was queried to identify lung cancer–related pathway maps containing MMP9, downstream target genes, and cancer-associated genetic markers. IPA was applied for pathway and functional enrichment analyses and causal reasoning to infer upstream regulators and downstream molecular effects. Cytoscape was used for network visualization and analysis, with high-confidence protein–protein interaction data imported via the STRING application within Cytoscape.

#### 4.1.2. Datasets

Datasets describing MMP9 involvement in lung cancer were generated by curated knowledgebase mining using MetaCore^TM^. Queries for “MMP9” (Homo sapiens) yielded 246 pathway maps, of which seven were lung cancer–specific. These maps were collated, and their gene and protein components were compared to identify shared nodes and recurrent functional modules, with overlap quantified using gene set overlap analyses.

To define downstream molecular consequences of MMP9 inhibition, curated outgoing interactions of MMP9 were extracted from MetaCore™, filtered for experimentally supported activation and inhibition relationships. In parallel, cancer-associated gene sets were assembled through advanced searches using the terms “apoptosis,” “migration,” and “NSCLC.” Intersection analysis identified overlapping genes shared across multiple cancer-related processes, representing core mediators of MMP9–associated signaling and prioritized for network reconstruction and biomarker signature nomination.

#### 4.1.3. Systems Biology Informatics Workflow

An integrative systems biology informatics approach [53] was employed to reconstruct MMP9 signaling networks and prioritize downstream molecular targets. Curated interactions from MetaCore^TM^ [50] and IPA [51] were filtered for lung cancer relevance and experimentally validated relationships. Networks were built using shortest-path algorithms centered on MMP9. Genes were prioritized based on six criteria: recurrence in lung cancer pathways, direct interaction with MMP9, association with apoptosis, association with migration, relevance to NSCLC relevance, and prior experimental evidence, generating a cumulative relevance score (0–6). IPA Core Analysis and biomarker filters were applied to nominate candidate biomarkers, while upstream regulator analysis inferred activation states using z-scores (|z| > 2) and Fisher’s exact test (*p* ≤ 0.05) [54]. Causal networks were visualized using IPA Path Designer to map regulatory relationships from MMP9 to functional outcomes.

### 4.2. Experimental Methods

#### 4.2.1. Compound Synthesis

Cinnamamide derivatives (M33–M41) were synthesized following an optimized synthetic protocol reported previously [34]. Full synthetic procedures are provided in the Supplementary Material Characterization of the chemical structures of the targeted compounds was performed using ^1^H-NMR, ^13^C-NMR, DEPT-135, and HRMS (ESI).

#### 4.2.2. MMP9 Enzyme Inhibition Assay

Inhibitory activity against purified MMP9 enzyme was evaluated as described previously [34], using N-isobutyl-N-(4-methoxyphenylsulfonyl)glycyl hydroxamic acid (NNGH) as a reference inhibitor. Compounds were incubated with MMP9 enzyme in assay buffer for 60 min at 37 °C, followed by addition of the chromogenic substrate Ac-PLG-[2-mercapto-4-methyl-pentanoyl]-LG-OC_2_H_5_. Absorbance at 412 nm was recorded every minute for 20 min to determine enzyme activity.

#### 4.2.3. Cell Viability Assay

Human lung cancer cell lines were obtained from ATCC. Cell viability was assessed using the MTT assay as previously described [53]. Cells were treated for 48, 72, and 96 h in RPMI medium. Experiments were performed in duplicate and repeated independently three times. IC_50_ values were calculated using nonlinear regression in GraphPad Prism 8 (GraphPad Software, Boston, MA, USA).

#### 4.2.4. Cell Migration Assay

Cell migration was evaluated using a wound healing assay as described previously [55]. A549 cells were seeded in Ibidi inserts and treated with compounds M1, M2, M8, M9, M10, M27, and M34 at IC_50_, ½IC_50_, and ¼IC_50_ concentrations. Images were acquired at 0 and 24 h using an EVOS XL Core imaging system (10×). Wound closure was quantified using ImageJ (v1.53e).

#### 4.2.5. Colony Formation Assay

Anchorage-independent growth was assessed using a soft agar clonogenic assay as previously described [6]. A549 cells were treated with compounds (M1, M2, M8, M9, M10, M27, and M34) at IC_50_, ½IC_50_, and ¼IC_50_ concentrations. Colonies were imaged after 12–14 days using an EVOS XL Core imaging system and quantified using ImageJ.

#### 4.2.6. Apoptosis Analysis

Apoptosis was assessed by Annexin V-FITC/propidium iodide (PI) staining. A549 cells were seeded at a density of 25 × 10^4^ cells per well in 6-well plates containing 5 mL of RPMI medium and incubated overnight at 37 °C in a humidified atmosphere (95% humidity) with 5% CO_2_ until reaching approximately 90% confluency, as described previously [56]. Cells were then treated with 2 × IC_50_ concentrations of selected compounds (M1, M2, M8, M9, M10, M27, and M34) for 48 h, with cisplatin used as a positive control. Following staining, samples were analyzed using a BD FACSCanto II flow cytometer (BD Biosciences, San Jose, CA, USA), and data were processed with BD FACSDiva software (v8.0).

#### 4.2.7. Cell Cycle Assay

Cell cycle distribution was assessed by PI staining following ethanol fixation [56]. A549 cells were treated with ½IC_50_ concentrations of compounds M1, M2, M8, M9, M10, M27, and M34 and incubated for 48 h at 37 °C in a humidified atmosphere containing 5% CO_2_ and 95% humidity. Following treatment, the cells were harvested, fixed overnight in 70% ice-cold ethanol at −20 °C and stained with PI, and analyzed immediately using a BD FACSCanto II flow cytometer (BD Biosciences, San Diego, CA, USA). The data were processed using BD FACSDiva software [6,56].

#### 4.2.8. Quantitative Real-Time Polymerase Chain Reaction (qRT-PCR)

A549, H1299, and H661 cells were seeded at a density of 4 × 10^5^ cells per 25 cm^2^ flask. A549 cells were treated with 0.1 × IC_50_ concentrations of selected compounds (M1, M2, M8, M9, M10, M27, and M34) for 72 h. Total RNA was extracted using the Direct-zol^TM^ RNA Miniprep Plus kit [53], quantified using a NanoDrop spectrophotometer, and reverse-transcribed with PrimeScript RT Master Mix (TaKaRa, Kusatsu, Japan) [56]. Quantitative real-time PCR (qRT-PCR) was performed on an ABI 7900 Real-Time PCR System using gene-specific primers (Supporting Information Table S1). Relative gene expression levels were calculated using the ΔΔCt method with GAPDH as the reference gene [57].

#### 4.2.9. Western Blot Analysis

MMP9 protein expression was evaluated by Western blotting as previously described [58]. A549 cells were treated with ½IC_50_ and ¼IC_50_ concentrations of selected compounds (M1, M2, M8, M9, M10, M27, and M34) for 72 h. Total protein lysates were separated by SDS-PAGE, transferred to nitrocellulose membranes, and probed with anti-MMP9 (1:20,000) (ThermoFisher Scientific, Waltham, MA, USA) and anti-GAPDH (1:10,000) (Abcam, Cambridge, UK) antibodies, followed by HRP-conjugated secondary antibodies.

#### 4.2.10. Statistical Analysis

Statistical analyses were performed using GraphPad Prism version 9 [59]. Data are presented as mean ± SD. Two-way ANOVA was applied for MTT assays, and one-way ANOVA for all other assays. A *p*-value ≤ 0.05 was considered statistically significant. IC_50_ values were estimated by nonlinear regression. ImageJ (v1.53e) [60] was used to quantify wound closure, colony formation, and protein band intensity.

## 5. Conclusions

Our integrative approach combining network biology, bioinformatics, and multi-level experimental validation provides a robust framework for target mechanism elucidation and pharmacodynamic biomarker nomination. Using purified enzyme assays, cellular phenotyping, and molecular validation, we identify MMP9 as a therapeutically relevant node in NSCLC and validate an eight-gene signature as a pharmacodynamic readout of MMP9 inhibition. This panel captures angiogenic, inflammatory, epithelial, and extracellular matrix-related processes downstream of MMP9. Beyond characterizing a new class of MMP9 inhibitors, this study establishes a generalizable workflow for mechanism-informed biomarker discovery and translational monitoring of targeted therapies in oncology.

## Figures and Tables

**Table 1:** Downstream targets of MMP9 identified via outgoing interaction analysis.

No.	Category	Downstream Target Genes	Mechanism
1	Growth Factors and Cytokines	*↑BMP7*, *↑TGFB1*, *↑TGFB2*, *↑TGFB3*, *↑VEGFA*, *↑IL1β*, *↑IL2*, *↑EMAP2*, *↓INS*, *↓PRL*, *↓IAPP*, *↓GRN*, *↓SERPINF1*, *↓SERPINE2*, *↓PLAT*, *↓APCS*, *↓A2M*	Cleavage
2	Chemokines (CC Family)	*↓* *CCL2* , *↓* *CCL7* , *↓* *CCL17* , *↓* *CCL11*	Cleavage
3	Chemokines (CXC Family)	*↑CXCL8*, *↓CXCL1*, *↓CXCL5*, *↓CXCL10*, *↓CXCL12*	Cleavage
4	Extracellular Matrix (ECM) and Structural Proteins	*↑* *SDC4* , *↑* *MAG* , *↓* *COL1A1* , *↓* *COL1A2* , *↓* *COL4A1* , *↓* *COL4A2* , *↓* *COL5A1* , *↓* *COL5A2* , *↓* *COL5A3* , *↓* *COL6A1* , *↓* *COL6A2* , *↓* *COL15A1* , *↓* *ELN* , *↓* *FBLN5* , *↓* *EMILIN1* , *↓* *EFEMP1* , *↓* *FN1* , *↓* *LTBP1* , *↓* *LTBP2* , *↓* *COMP* , *↓* *VCAN* , *↓* *VTN* , *↓* *HSPG2* , *↓* *SDC1* , *↓* *SPOCK3* , *↓* *LAMB1* , *↓* *NID*	Cleavage
5	Cell Adhesion and Tight Junction Proteins	*↓* *CDH1* , *↓* *CLDN5* , *↓* *OCLN* , *↓* *TJP1* , *↓* *CAP1* , *↓* *ADGRA2*	Cleavage
6	Signaling Receptors	*↑* *EPHB2* , *↓* *KIT* , *↓* *FLT1* , *↓* *NTRK2*	Cleavage
7	Binding and Regulatory Proteins	*↓* *IGFBP1* , *↓* *IGFBP3* , *↓* *IGFBP6* , *↓* *APOA1* , *↓* *LGALS1* , *↓* *LGALS3* , *↓* *TFP1*	Cleavage
8	Transcriptional Regulators	*↑* *ETV4*	Influence on expression
		*↑VIM*, *↓CTNNB1*	Cleavage
9	Enzymes and Enzyme Regulators	*↓* *TIMP1* , *↓* *TIMP2* , *↓* *MYLK3*	Cleavage
10	Other proteins	*↑ADGRA2*, *↑SCUBE3*, *↑EDN1*, *↓APCS*, *↓SFTPD*, *↓OPTC*, *↓APP*, *↓EMAP2*, *↓AEBP1*	Cleavage

Red indicates activation, green indicates inhibition, *↑ indicates up-regulation, ↓ indicates down-regulation*.

**Table 2:** Prioritized gene targets for MMP9 network analysis in NSCLC.

Gene	Direct Interaction (Ref)	Lung Cancer	Apoptosis	Migration	NSCLC	Experimental Evidence	Total Score
*APP*	1 [18]	0	1	1	1	1	5
*CCL17*	1 [19]	0	1	1	1	1	5
*CCL2*	1 [20]	0	1	1	1	1	5
*CDH1*	1 [21]	1	0	1	1	1	5
*COL5A1*	1 [22]	0	1	1	1	1	5
*COL6A2*	1 [23]	0	1	1	1	1	5
*VEGFA*	1 [24]	1	0	1	1	1	5
*ETV4*	0	0	1	1	1	1	4
*A2M*	1 [25]	0	0	0	1	1	3
*APOA1*	1 [26]	0	0	1	0	1	3
*BMP7*	1 [27]	0	1	0	0	1	3
*CAP1*	1 [28]	0	0	1	0	1	3
*COL6A1*	1 [29]	0	1	0	0	1	3
*COMP*	1 [30]	0	1	0	0	1	3
*IL1B*	1 [31]	1	0	0	0	1	3
*LAMB1*	1 [32]	0	0	1	0	1	3
*VIM*	1 [33]	1	0	0	0	1	3
*CTNNB1*	0	1	0	0	0	1	2

Genes were ranked based on a cumulative score derived from six criteria: MMP9 interaction, overlap with lung cancer, apoptosis, migration, and NSCLC gene sets, and experimental validation (1 = yes, 0 = no). Direct interaction references are indicated in brackets.

**Table 3:** The top 10 enriched canonical pathways from IPA core analysis of MMP9–associated genes.

Enriched Pathways	FDR	Overlapping Genes
Pathogen-Induced Cytokine Storm Signaling Pathway	6.03 × 10^−9^	*CCL17*, *CCL2*, *CDH1*, *COL5A1*, *COL6A2*, *VEGFA*
Hepatic Fibrosis/Hepatic Stellate Cell Activation	1.02 × 10^−8^	*CCL2*, *COL5A1*, *COL6A2*, *MMP9*, *VEGFA*
Hepatitis B Chronic Liver Pathogenesis Signaling Pathway	8.98 × 10^−7^	*CCL2*, *ETV4*, *MMP9*, *VEGFA*
IL-17 Signaling Pathway	6.88 × 10^−7^	*CCL17*, *CCL2*, *MMP9*, *VEGFA*
Glycation Signaling Pathway	1.10 × 10^−6^	*APP*, *CCL2*, *MMP9*, *VEGFA*
Wound Healing Signaling Pathway	1.39 × 10^−6^	*COL5A1*, *COL6A2*, *MMP9*, *VEGFA*
Assembly of collagen fibrils and other multimeric structures	1.27 × 10^−6^	*COL5A1*, *COL6A2*, *MMP9*
Collagen degradation	1.28 × 10^−6^	*COL5A1*, *COL6A2*, *MMP9*
Role of Osteoclasts in Rheumatoid Arthritis Signaling Pathway	2.38 × 10^−6^	*CDH1*, *COL5A1*, *COL6A2*, *MMP9*
S100 Family Signaling Pathway	2.24 × 10^−6^	*APP*, *CDH1*, *ETV4*, *MMP9*, *VEGFA*

Analysis was conducted using IPA. The FDR (False Discovery Rate) reflects the statistical significance of the enrichment.

**Table 4:** Effect of MMP9 inhibitors on the expression of *MMP9* and downstream targets in A549 lung cancer cells.

Genes	Compounds						
	M1	M2	M8	M9	M10	M27	M34
*MMP9*	−3.84	−3.64	−3.47	−2.84	−1.60	−3.06	−3.64
*APP*	−4.32	−1.47	−1.89	−0.94	−0.32	−0.79	−1.89
*ETV4*	−3.06	−4.06	−2.94	−2.39	−2.18	−1.69	−3.18
*VEGFA*	−2.00	−2.64	−2.94	−3.47	−1.15	−0.76	−2.25
*COL5A1*	+3.15	+3.75	+2.46	+1.15	+1.19	+1.98	+2.12
*COL6A2*	+2.10	+2.58	+0.77	+0.68	+1.31	+0.85	+0.69
*CCL2*	+3.07	+5.13	+2.04	+2.70	+2.82	+1.48	+3.77
*CCL17*	+0.15	+0.29	+0.32	+0.15	+0.19	+0.49	+0.11
*CDH1*	+0.40	+1.81	+0.89	+0.39	+1.35	+0.15	+1.54

A549 cells were treated with MMP9 inhibitors at 0.1 × IC_50_. Relative mRNA expression levels were quantified by real-time quantitative PCR and expressed as fold change (2^−ΔΔCt^) relative to untreated control, with the corresponding percent change versus control shown in parentheses. Fold-change values were additionally transformed to log_2_ scale (log_2_FC = log_2_[treated/control]) and are presented in the table. GAPDH was used as the internal housekeeping gene for normalization. Data represent the mean of three independent experiments performed in duplicate. All values are statistically significant compared with the control (*p* ≤ 0.05). Fold-change values < 1 indicate decreased expression, whereas values > 1 indicate increased expression.

**Table 5:** Integrated experimental validation, upstream regulator analysis, and biomarker assessment of MMP9 inhibition in A549 NSCLC cells.

Gene	Predicted Effect for MMP9 Inhibition	Experimental Results	Matched Direction	Biological Meaning	Upstream Regulators (Z-Score)	Readout Type	Biomarker for Lung Cancer	Biomarker for MMP9 Inhibition
*VEGFA*	Downregulation	Downregulation	Yes	Angiogenesis	PKC (−1.000) NORAD (2.000) INHBA (−0.577) SERPINB7 (0.577)	mRNA Protein	Yes	No
*CDH1*	Upregulation	Upregulation	Yes	EMT reversal/cell adhesion	TERC (−1.732) TNFSF12 (−0.577) SNAI1 (−0.577) FBN2 (−1.414)	mRNA Protein	Yes	No
*CCL2*	Upregulation	Upregulation	Yes	Immune cell recruitment	PKC (−1.000) TNFSF12 (−0.577) CHIA (1.414) INHBA (−0.577) SERPINB7 (0.577)	mRNA ELISA	Yes	No
*CCL17*	Upregulation	Upregulation	Yes	Immune chemotaxis	SNAI1 (−0.577) CHIA (1.414) INHBA (−0.577) CDH1 (0.000)	mRNA ELISA	No	No
*ETV4*	Downregulation	Downregulation	Yes	Invasion/EMT transcription factor	-	mRNA	Yes	No
*COL5A1*	Upregulation	Upregulation	Yes	ECM restoration	NORAD (2.000) TERC (−1.732)	mRNA Protein	No	No
*COL6A2*	Upregulation	Upregulation	Yes	ECM restoration	NORAD (2.000)	mRNA Protein	No	No
*APP*	Context-dependent	Downregulation	No	Proteolysis/stress signaling	PKC (−1.000) NORAD (2.000)	mRNA Protein	No	No

Upstream regulators were inferred using Ingenuity Pathway Analysis (IPA) based on transcriptional responses to compounds M1, M2, M8, M9, M10, M27, and M34. The Z-score for upstream regulators indicates predicted regulator activity (positive = activation, negative = inhibition; 0 = undetermined). Matched direction indicates concordance between predicted and experimentally observed gene expression changes.

## Data Availability

The original contributions presented in this study are included in the article/Supplementary Material. Further inquiries can be directed to the corresponding authors.

## Supplementary Materials

The following supporting information can be downloaded at: https://www.mdpi.com/article/10.3390/ijms27146457/s1.

## Abbreviations

µM	Micromolar
A2M	Alpha-2-Macroglobulin
APOA1	Apolipoprotein A1
APP	Amyloid Precursor Protein
BMP7	Bone Morphogenetic Protein 7
CAP1	Adenylyl Cyclase-Associated Protein 1
CCL17	C-C Motif Chemokine Ligand 17
CCL2	C-C Motif Chemokine Ligand 2
CDH1	Cadherin-1
CLDN5	Claudin-5
COL5A1	Collagen Type V Alpha 1 Chain
COL6A2	Collagen Type VI Alpha 2 Chain
CTNNB1	Catenin Beta-1
CXCL8	C-X-C Motif Chemokine Ligand 8
ECM	Extracellular Matrix
EGFR	Epidermal Growth Factor Receptor
ELN	Elastin Gene
EMT	Epithelial–Mesenchymal Transition
ETV4	E-twenty-six (ETS) Variant Transcription Factor 4
FBN2	Fibrillin 2
FDR	False Discovery Rate
FN1	Fibronectin 1
GAPDH	Glyceraldehyde-3-Phosphate Dehydrogenase
HGF	Hepatocyte Growth Factor
IC50	Half-Maximal Inhibitory Concentration
IGF	Insulin-like Growth Factor
IL-17	Interleukin-17
IL1B	Interleukin 1 Beta
INHBA	Inhibin Subunit Beta A
IPA	Ingenuity Pathway Analysis
KRAS	Kirsten Rat Sarcoma Viral Oncogene Homolog
LAMB1	Laminin Subunit Beta 1
MET	MET Proto-Oncogene
MMP1	Matrix Metalloproteinase-1
MMP9	Matrix Metalloproteinase-9
NF-Κb	Nuclear Factor kappa-light-Chain-Enhancer of Activated B Cells
NNGH	N-isobutyl-N-(4-methoxyphenylsulfonyl)glycyl Hydroxamic Acid
NSCLC	Non-Small Cell Lung Cancer
PD	Pharmacodynamic
PKC	Protein Kinase C
PPI	Protein–Protein Interaction
qRT-PCR	Quantitative Reverse Transcription Polymerase Chain Reaction
SD	Standard Deviation
SERPINB7	Serpin Family B Member 7
SNAI1	Snail Family Transcriptional Repressor 1
TERC	Telomerase RNA Component
TGF-β	Transforming Growth Factor beta
THBS1	Thrombospondin 1
TIMP1–3	Tissue Inhibitor of Metalloproteinases
TNFSF12	Tumor Necrosis Factor (ligand) Superfamily, Member 12
VEGFA	Vascular Endothelial Growth Factor A
VIM	Vimentin
WNT	Wingless-Related Integration Site
