# Supplementary material for: Systems Biology and Experimental Validation Enable Discovery of MMP9-Centered Networks, Anticancer Activity, and Pharmacodynamic Signature in Non-Small Cell Lung Cancer
Source: Int J Mol Sci. 2026 Jul 20;27(14):6457. doi: 10.3390/ijms27146457 (PMC13410491; doi:10.3390/ijms27146457)
Supplement: Supplementary file 1 [file ijms-27-06457-s001.zip › ijms-4413530-supplementary.pdf]

Systems Biology and Experimental Validation Enable Discovery of MMP9-Centered Networks, Anticancer Activity, and Pharmacodynamic Signature in Non-Small Cell Lung Cancer

Zainab Ahmed Rashid[1], Rima Hajjo[2,3*], Dima A. Sabbah[2], Kamal Sweidan[4], Shriefa Almutairi[1], and Sanaa K. Bardaweel[1*]

[1] Department of Pharmaceutical Sciences, School of Pharmacy, The University of Jordan, Amman 11942, Jordan; zainab.rashid1995@gmail.com (Z.A.R.); shriefaalmutairi@gmail.com (S.A.)

[2] Department of Pharmacy, Faculty of Pharmacy, Al-Zaytoonah University of Jordan, P.O. Box 130, Amman 11733, Jordan; dima.sabbah@zuj.edu.jo

[3] Laboratory for Molecular Modeling, Division of Chemical Biology and Medicinal Chemistry, Eshelman School of Pharmacy, The University of North Carolina at Chapel Hill, Chapel Hill, NC 27599, USA

[4] Department of Chemistry, The University of Jordan, Amman 11942, Jordan; k.sweidan@ju.edu.jo

*Correspondence: r.hajjo@zuj.edu.jo (R.H.); s.bardaweel@ju.edu.jo (S.K.B.)

Short Title: MMP9 Inhibition and Biomarker Discovery in Lung Cancer

Supplementary Materials

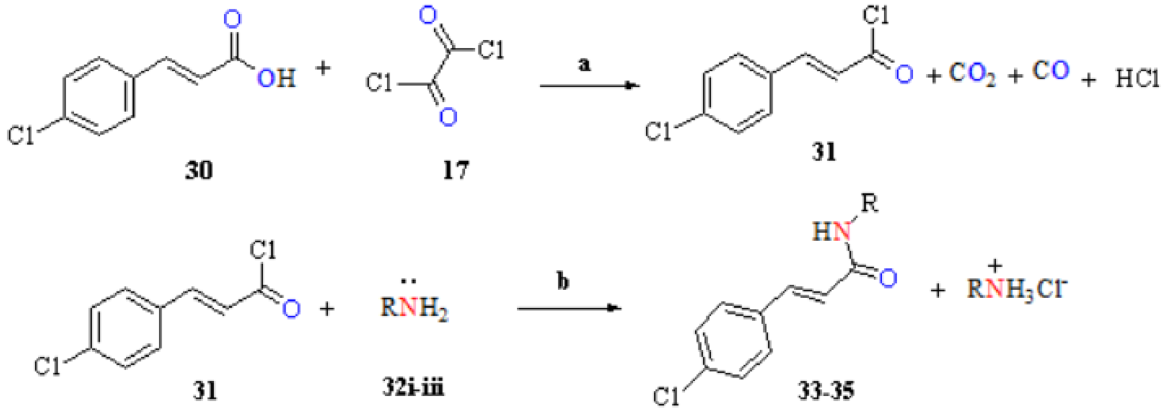

Scheme S1. Synthesis of *p*-Chlorocinnamamide Derivatives. Reagents and conditions: (**a**) (1) CHCl3, DMF, 0°C, 30 min, (2) 80°C, 2 hours (**b**) CHCl3, pyridine, 80ºC, 24hr.

The chemical structures of *p*-chlorocinnamamide derivatives.

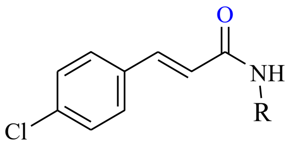

No.	Code	R
1	M33	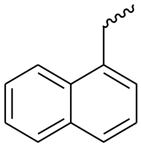
2	M34	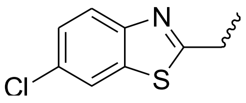
3	M35	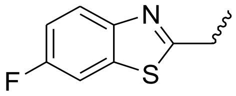

Figures S1A -C. The ^{1}H NMR, ^{13}C NMR, and DEPT ^{13}C-NMR spectra of **M33**

- **(E)-3-(4-chlorophenyl)-*N*-(naphthalen-1-yl) acrylamide (M33)**

Brown powder; yield (70 %); Mp 215-218 °C; mobile phase (EtOAc: n-hexane)(1:3); Rf = 0.25; **^{1}H-NMR (500 MHz, DMSO-d$_6$)** δ (ppm): 6.3 (d, J = 16.0 Hz, 1H, H2), 6.7 (d, J = 7.5 Hz, 1H, Ar-H2''), 6.8 (ps. d, 1H, Ar-H4''), 6.9-6.9 (m, 3H, Ar-H5'' + H6''+ H7''), 7.0 – 7.2 (m, 3H, H3+ Ar-H3''+ H8''), 7.3 – 7.4 (d, 2H, J = 8.6 Hz, Ar-H2'/ H6'), 7.5 (d, 2H, J = 8.6 Hz, Ar-H3'/ H5') ppm. **^{13}C-NMR (125 MHz, DMSO-d$_6$)** δ (ppm): 109.3 (C2''), 110.7 (C2), 115.3 (C4''), 118.8 (C8''), 119.6 (C7''), 121.0 (C8''a), 125.7 (C3''), 126.0 (C2' / C6'), 126.6 (C5''), 131.4 (C1'), 136.4 (C4'), 143.8 (C4''a), 145.9 (C5' / C3'), 153.8 (C1''), 158.1 (C3), 169.8 (C1) ppm.

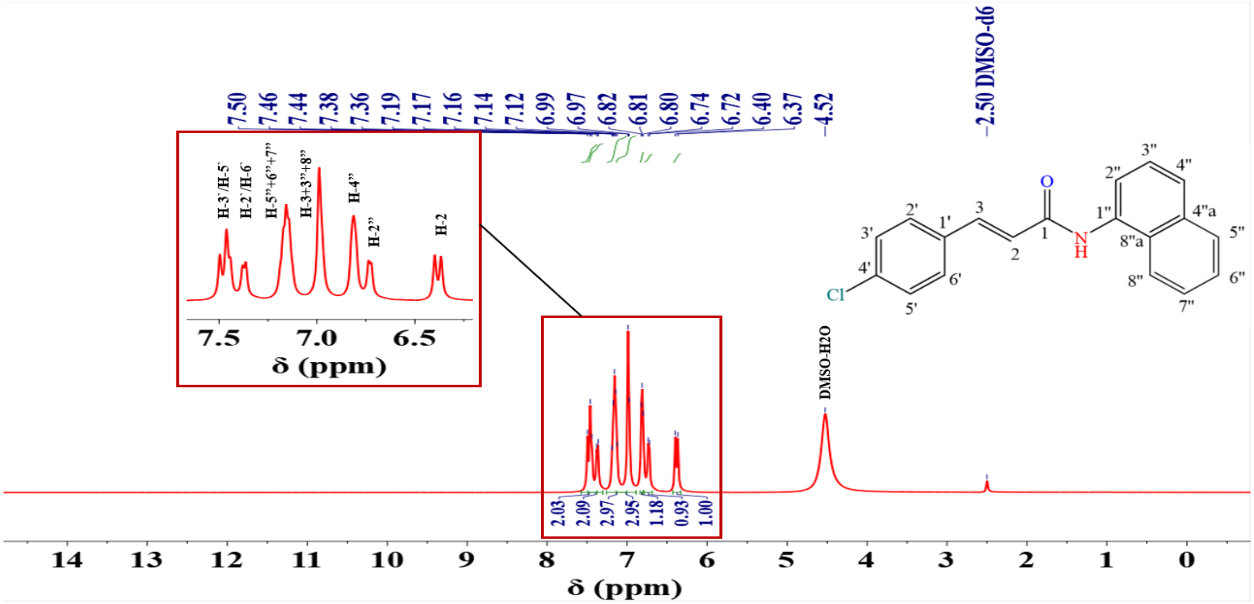

Figure S1A. The ^{1}H NMR spectrum of compound M33. ^{1}H NMR instrument (500 MHz), solvent used is (DMSO-d6).

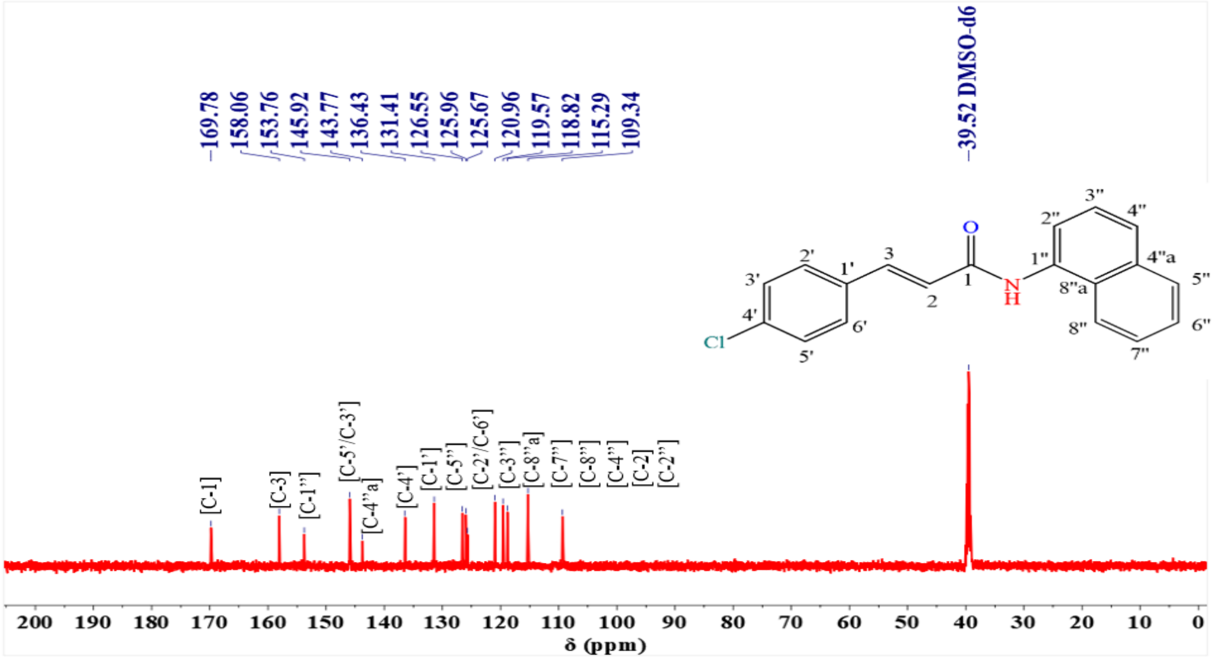

Figure S1B. The ^{13}C NMR spectrum of compound M33. NMR instrument (500 MHz), solvent used is (DMSO-d6)

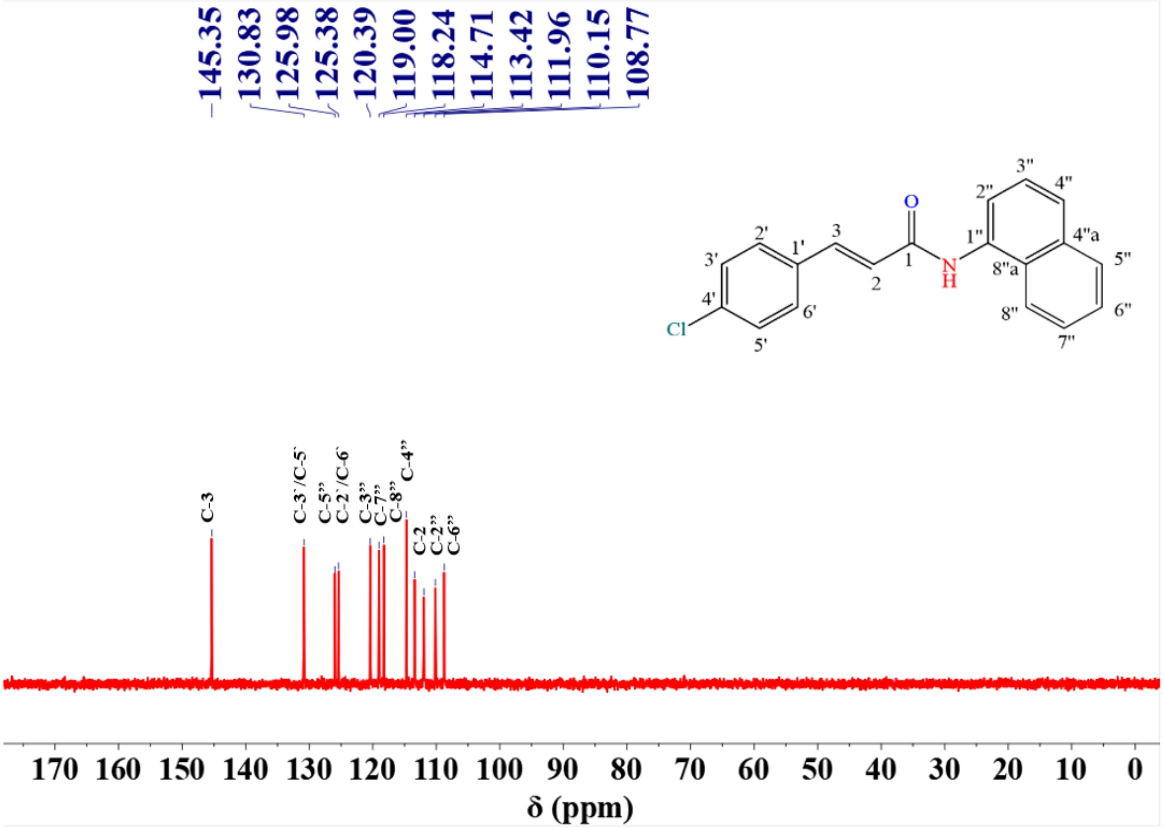

Figure S1C. The DEPT ^{13}C-NMR spectrum of M33.

Figures S2A-C. The ^{1}H NMR, ^{13}C NMR, and DEPT ^{13}C-NMR spectra of M34

- **(E)-*N*-(6-chlorobenzo(d)thiazol-2-yl)-3-(4-chlorophenyl) acrylamide (M34)**

Brown powder; yield (72%); Mp 143-146 °C; mobile phase (EtOAc: n-hexane)(1:3); Rf = 0.18; **^{1}H-NMR (500 MHz, DMSO-d$_6$)** δ (ppm): 6.2 (d, J = 16.0 Hz, 1H, H2`), 6.7 (br. d, 2H, Ar-H3''/ H5''), 6.9 (br. t, 3H, Ar-H7 + H5+ H4), 7.0-7.1 (m, 2H, Ar-H2''/H6''), 7.4 (d, $J$ = 16.1 Hz, 2H, H3`) ppm. **^{13}C-NMR (125 MHz, DMSO-d$_6$)** δ (ppm): 115.5 (C4), 119.0 (C2'' / C6''), 119.5 (C4'' + C6), 121.3 (C5''/C3''), 121.8 (C5), 127.4 (C7), 131.5 (C7a), 136.6 (C1''), 146.3 (C2`), 150.6 (C3'), 158.0 (C3a), 169.5 (C1'), 170.3 (C2) ppm.

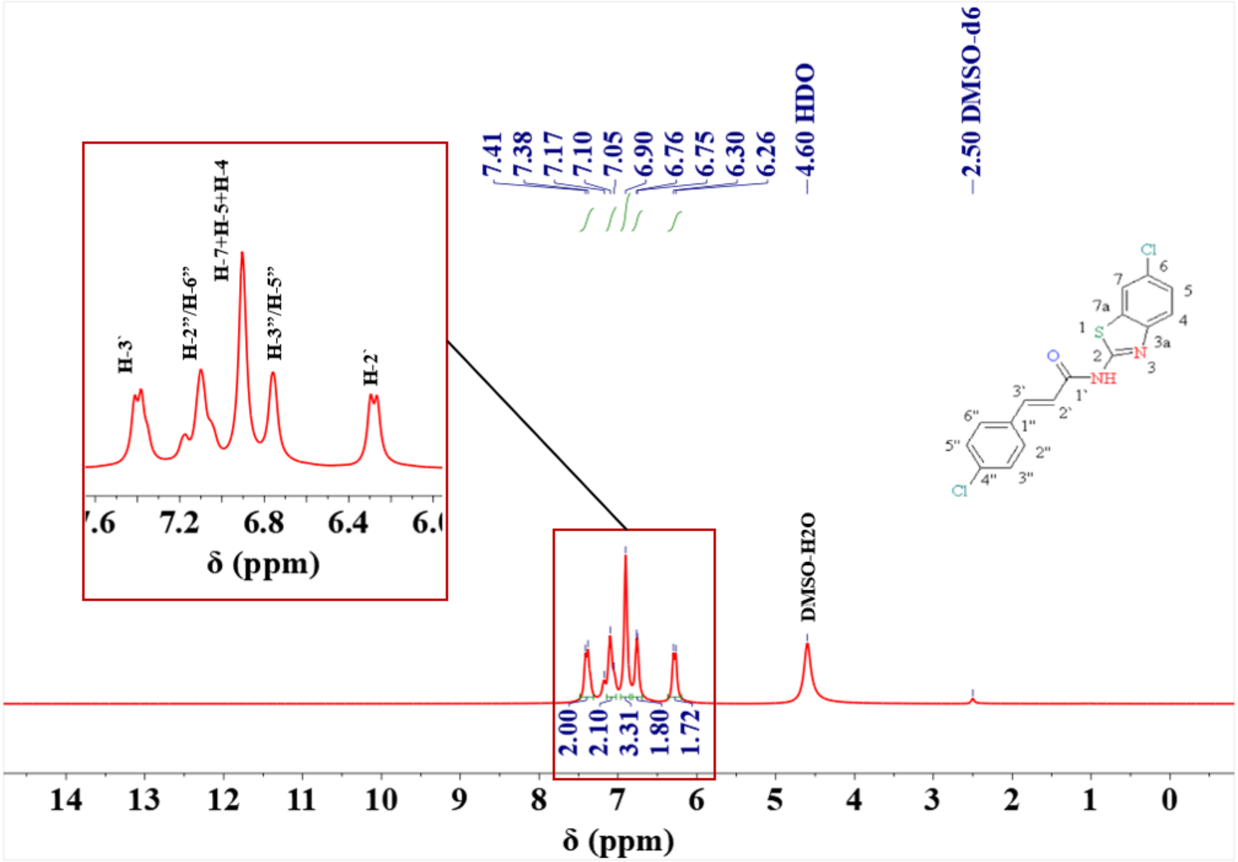

Figure S2A. The ^{1}H NMR spectrum of compound M34. ^{1}H NMR instrument (500 MHz), solvent used is (DMSO-d6).

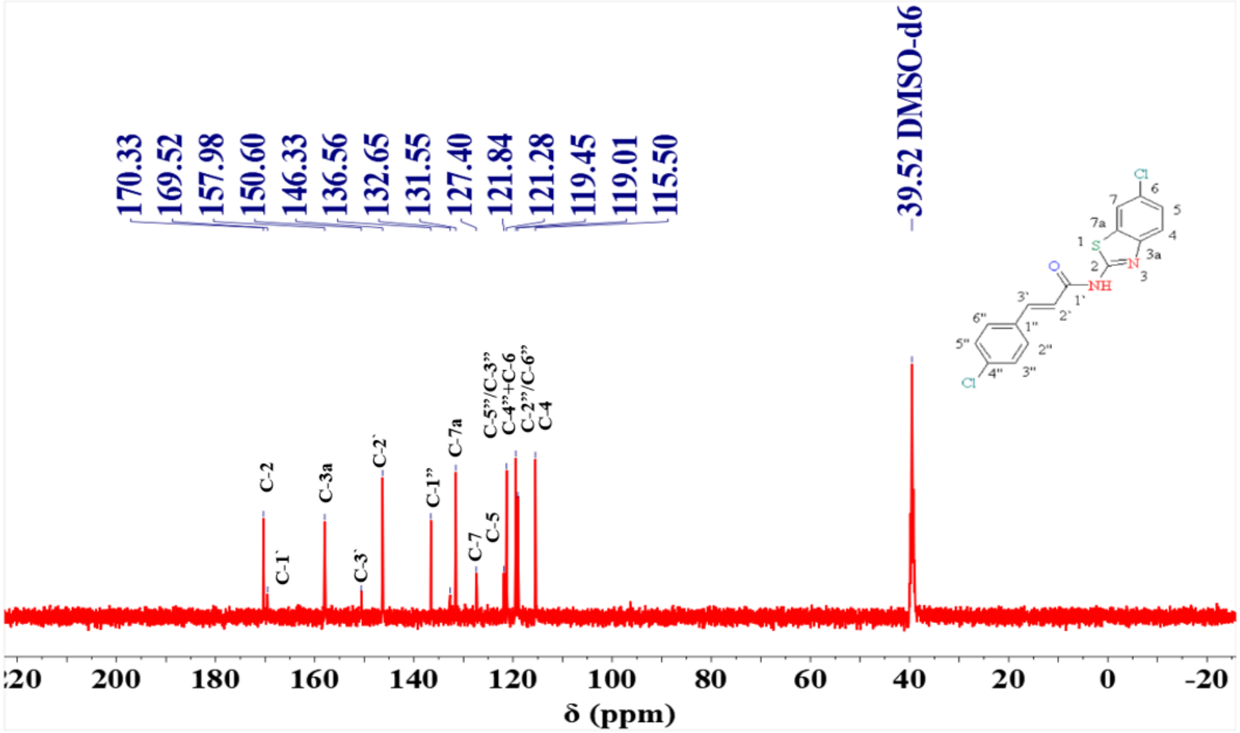

Figure S2B. The ^{13}C NMR spectrum of compound M34. NMR instrument (500 MHz), solvent used is (DMSO-d6)

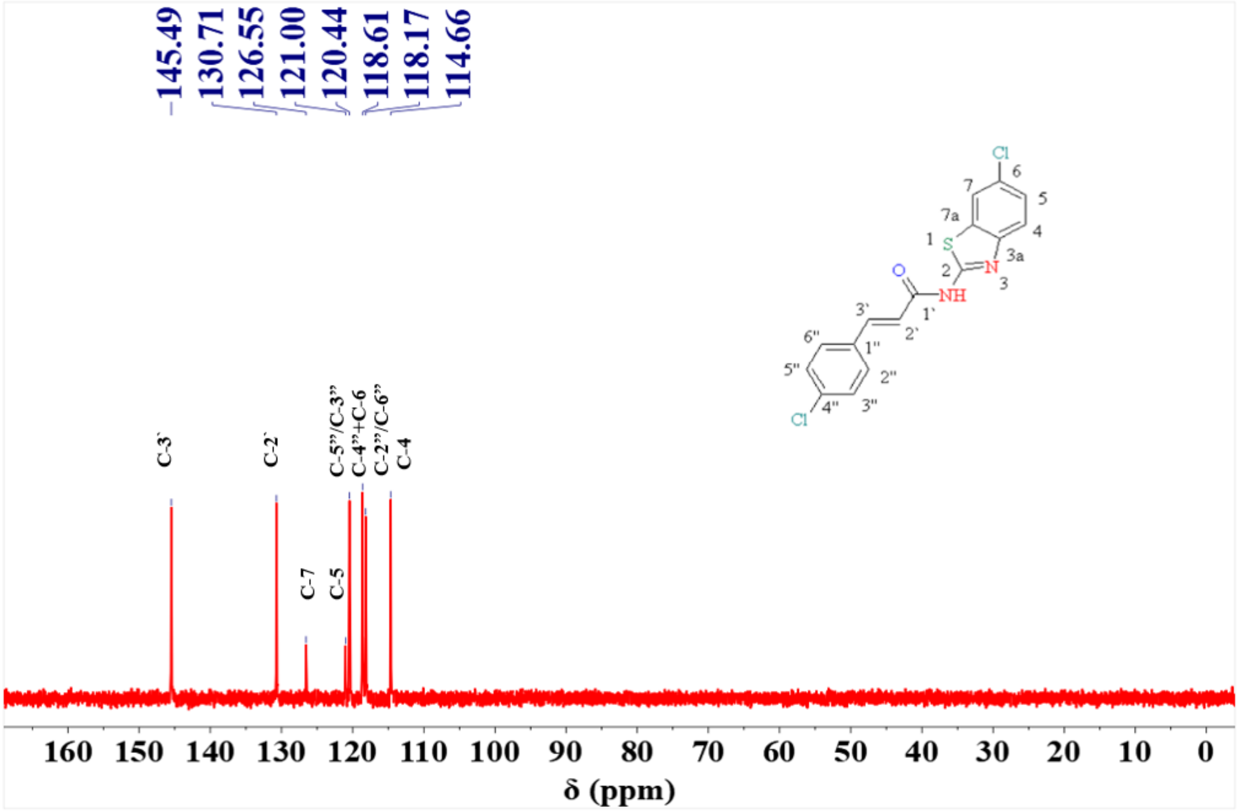

Figure S2C. The DEPT ^{13}C-NMR spectrum of M34.

Figures S3A-B. The ^{1}H NMR and ^{13}C NMR spectra of M35

- **3-(4-Chloro-phenyl)-*N*-(6-fluoro-benzothiazol-2-yl)-acrylamide (M35)**

Brown powder; yield (68 %); Mp 169-174 °C, mobile phase (EtOAc: n-hexane) (1:3); Rf = 0.33; **^{1}H-NMR (500 MHz, DMSO-d$_6$)** δ (ppm): 6.5 (d, J = 16.0 Hz, 1H, H2\`), 7.4 (d, $J$ = 8.5 Hz, 2H, Ar-H2''/H6''), 7.5 (d, $J$ = 16.0 Hz, 1H, H3\`), 7.7 (d, J = 8.6 Hz, 2H, Ar-H3''/H5''), 8.0 – 8.1 (t, 1H, Ar-H5), 8.9 – 8.9 (d, 1H, Ar-H4), 9.5 (s, 1H, NH amide).**^{13}C-NMR (125 MHz, DMSO-d$_6$)** δ (ppm): 108.3-108.5 (C7), 109.6-109.8 (C5), 114.4 (C2\`),115.9 -115.9 (C4), 120.1 (C7a), 127.2 (C2''/C6''), 128.9 (C3''/C5''), 129.9 (C1''), 133.2 (C4''), 134.7 (C3\`), 138.5 (C3a), 142 (C6), 160.9 (C6), 167.4 (C1\`), 168.5 (C2).

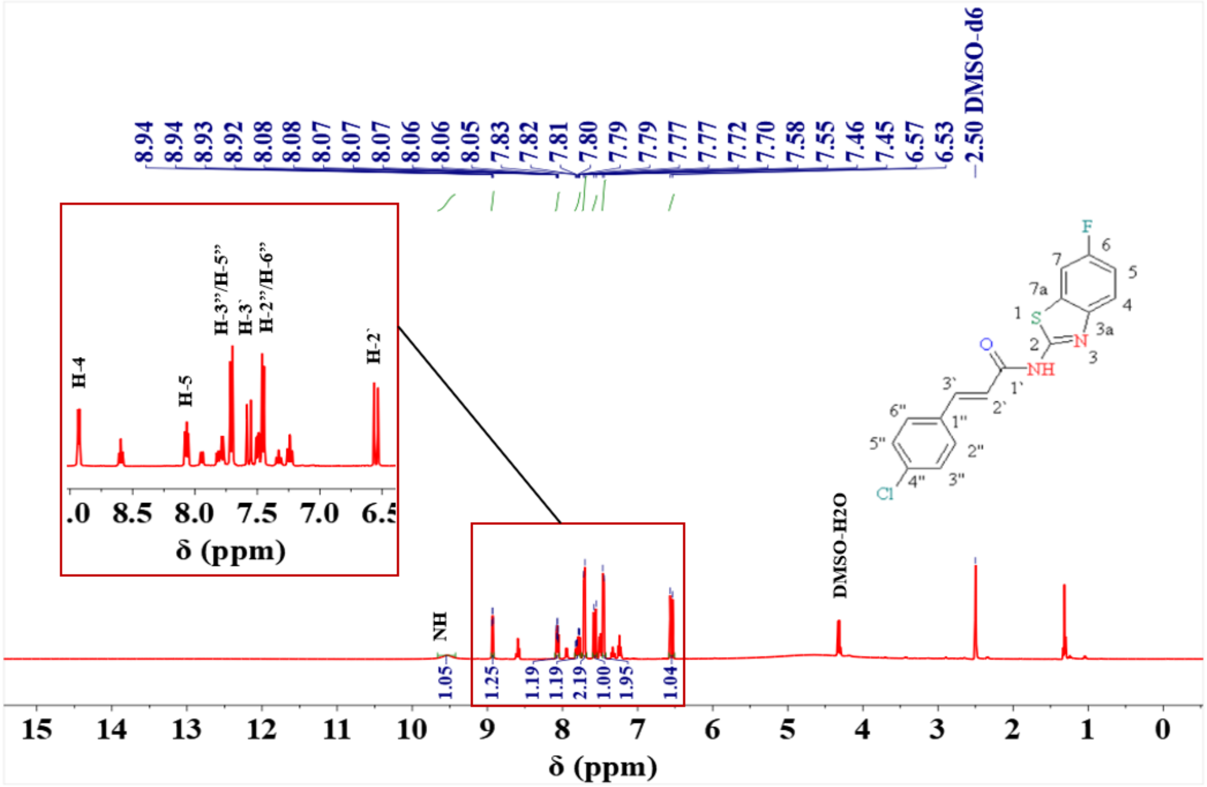

Figure S3A. The ^{1}H NMR spectrum of compound M35. ^{1}H NMR instrument (500 MHz), solvent used is (DMSO-d6).

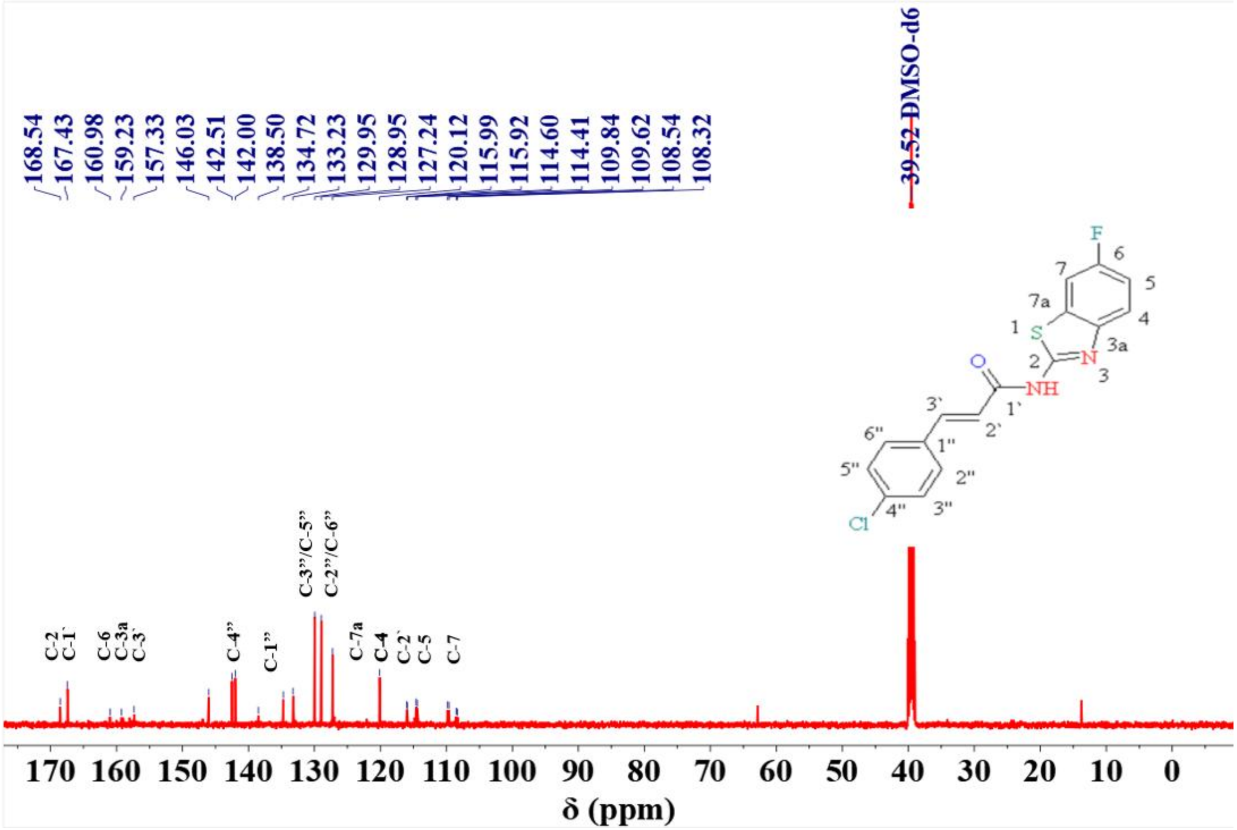

Figure S3B. The ^{13}C NMR spectrum of compound M35. NMR instrument (500 MHz), solvent used is (DMSO-d6).

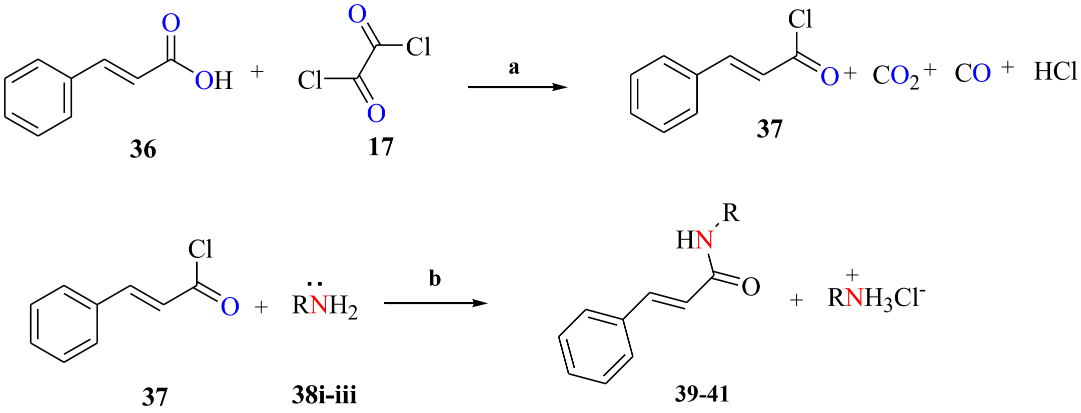

Scheme S2. Synthesis of cinnamamide. Reagents and conditions: (**a**) (1) $CHCl_3$, DMF, 0°C, 30 min, (2) 80°C, 2 hours (**b**) $CHCl_3$, pyridine, 80ºC, 24hr.

The chemical structures of cinnamamide derivatives.

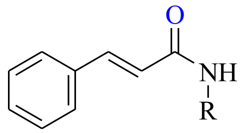

No.	Code	R
1	M39	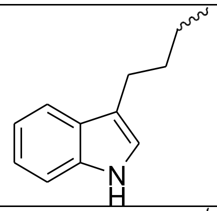
2	M40	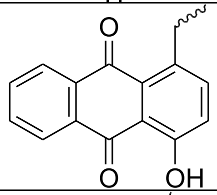
3	M41	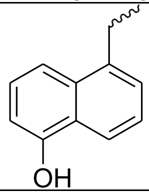

Figures S4 A-C. The ^{1}H NMR, ^{13}C NMR, and DEPT ^{13}C-NMR spectra of M39

- ***N*-(2-(1H-Indol-3-yl)-ethyl)-3-phenyl-acrylamide** (**M39**)

Faint yellow powder; yield (65%); Mp 115-118 °C, mobile phase (Column chromatography) (EtOAc: n-hexane) (30:70); Rf = 0.42; **^{1}H-NMR (500 MHz, DMSO-d$_6$)** δ (ppm): 3.1 (s,2H, H1`), 3.3 (s, 2H, H2`), 7.2 (d, J = 15.5 Hz, 2H, Ar-H6''/H8''), 7.3 – 7.4 (m, 4H, Ar-H5/H6 +H2+H7''),

7.4 (d, *J* = 15.4 Hz, 2H, Ar-H5’’/H9’’), 7.6 – 7.7 (m, 4H, H-3’’+H-2’’+Ar-H4+H7).**^{13}C-NMR (125 MHz, DMSO-d$_6$)** δ (ppm): 35.3 (C2`), 36.8 (C1`), 118.5 (C3), 127.4 (C2’’), 127.9 (C2), 128.7 (C4/C7), 128.9 (C7’’), 129.4 (C5/C6),135.1 (C5’’/C9’’), 140.9 (C6’’/C8’’), 165.5 (C1’’).

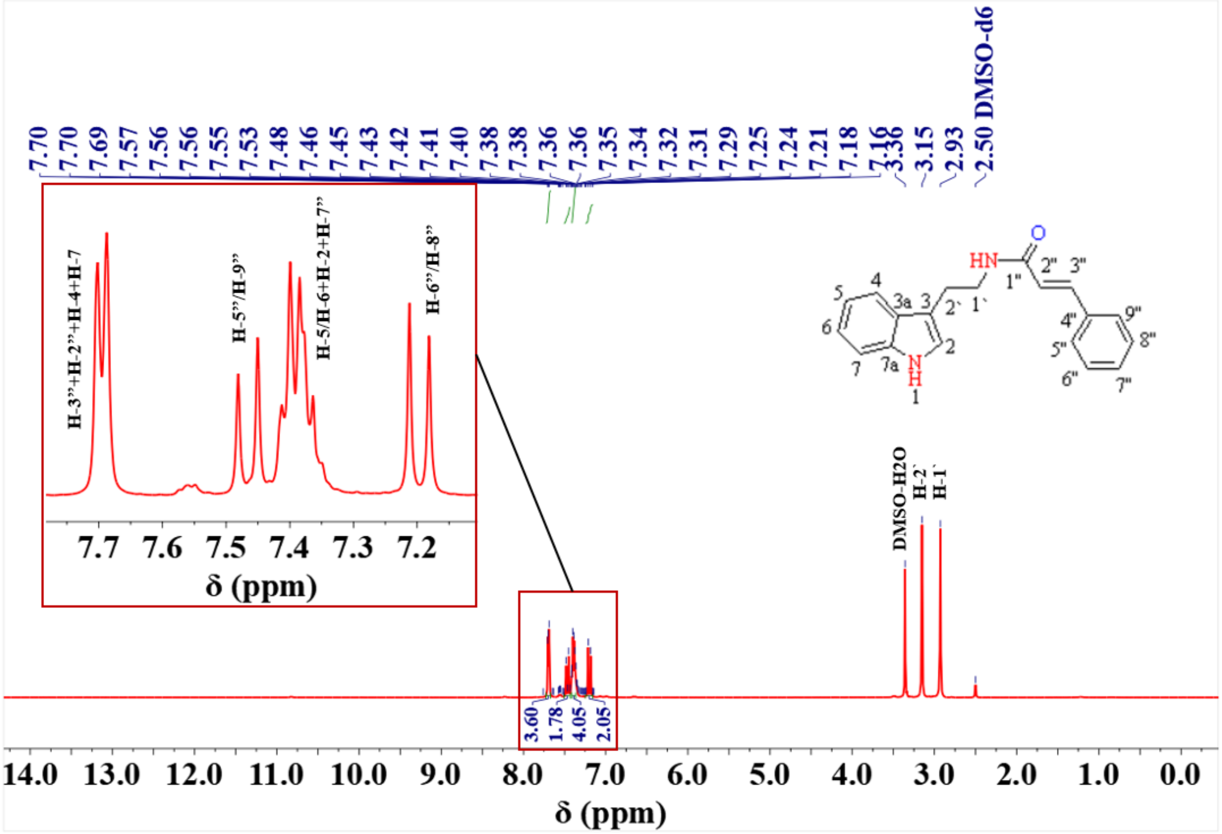

Figures S4A. The ^{1}H NMR spectrum of compound M39. ^{1}H NMR instrument (500 MHz), solvent used is (DMSO-d6).

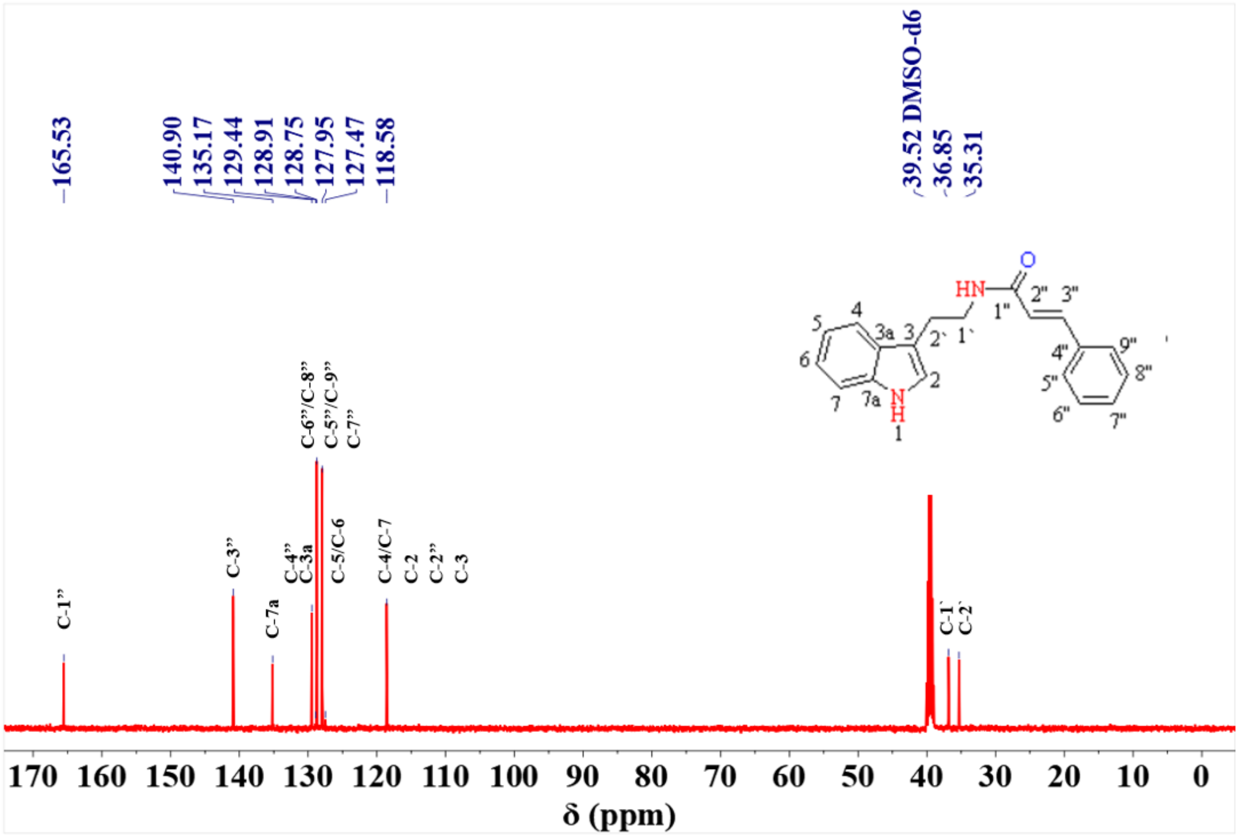

Figures S4B. The ^{13}C NMR spectrum of compound M39. NMR instrument (500 MHz), solvent used is (DMSO-d6).

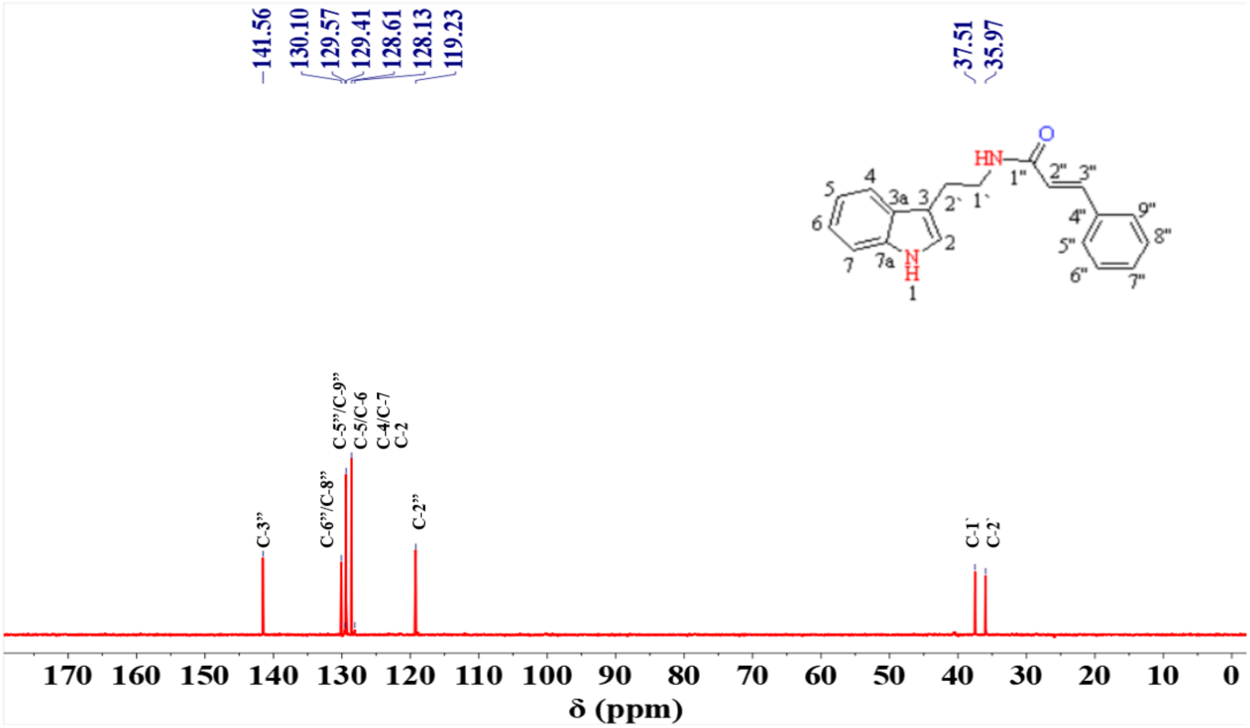

Figures S4C. The DEPT ^{13}C-NMR spectrum of M39.

Figures S5A-C. The ^{1}H NMR, ^{13}C NMR, and DEPT ^{13}C-NMR spectra of M40.

- ***N*-(4-Hydroxy-9,10-dioxo-9,10-dihydro-anthracen-1-yl)-3-phenyl-acrylamide** (**M40**)

Orange powder; yield (62 %); Mp 177-180 °C; mobile phase (Column chromatography) (EtOAc: n-hexane) (10:90); Rf = 0.47; **^{1}H-NMR (500 MHz, DMSO-d_6)** δ (ppm): 7.0 (d, J = 16.1 Hz, 1H,H2`), 7.5 (t, 2H, Ar-H7/H8), 7.7 (d, $J$ = 9.4 Hz, 1H, H3`), 7.8 (d, J = 7.9 Hz, 2H, Ar-H2+H3), 7.8 – 7.9 (m, 3H, Ar-H3``/H5``+H4``), 8.0 (d, $J$ = 7.5 Hz, 2H, Ar-H6/H9), 8.1 (d, $J$ = 7.5 Hz, 2H, Ar-H2``/H6``).**$^{13}C$-NMR (125 MHz, DMSO-$d_6$)** $\delta$ (ppm): 117.7 (C3), 125.66 (C2`), 126.8 (C2), 129.2 (C4a), 129.6 (C2``/C6``), 131.5 (C3``/C5``), 133.4 (C6/C9), 133.6 (C7/C8), 134.4 (C4``), 135.1 (C1), 135.3 (C10a), 135.6 (C1``), 138.4 (C5a/C9a), 140.2 (C3`), 147.2 (C4), 159.8 (C1`), 165.4 (C5/C10).

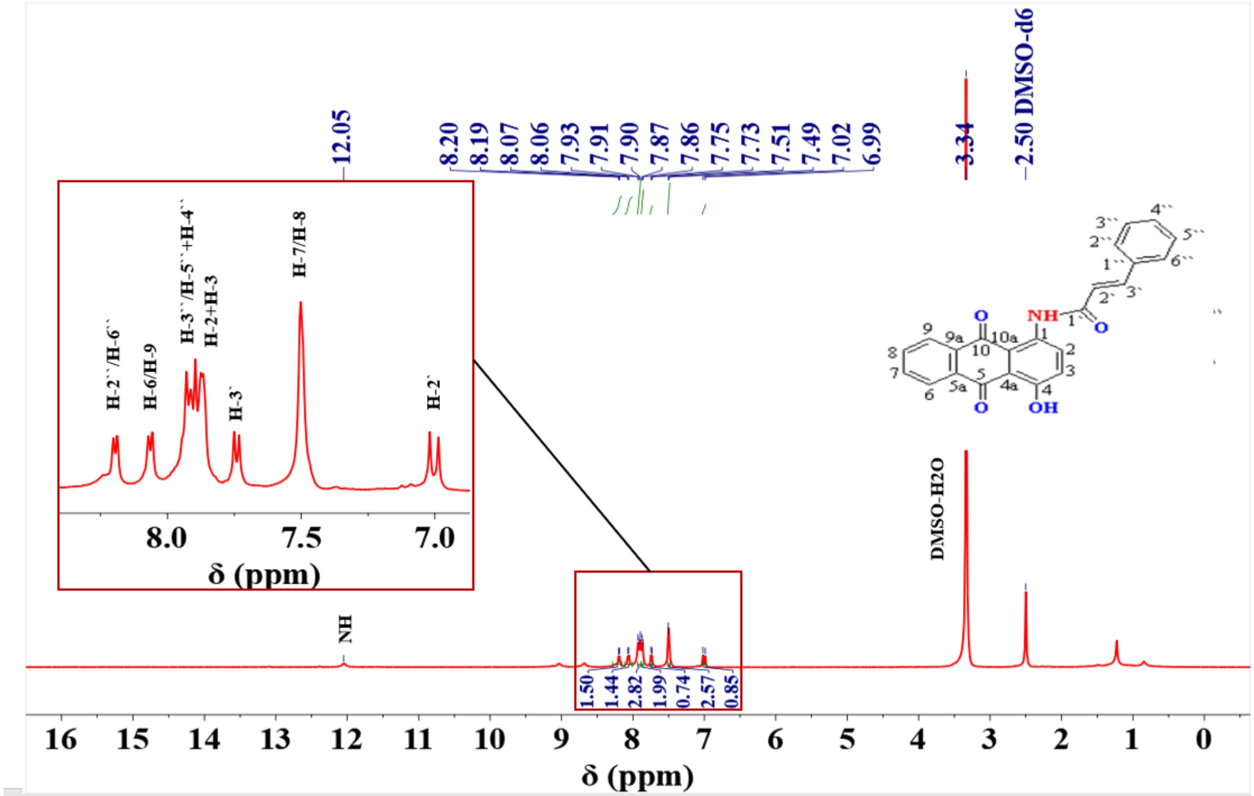

Figure S5A. The ^{1}H NMR spectrum of compound M40. ^{1}H NMR instrument (500 MHz), solvent used is (DMSO-d6).

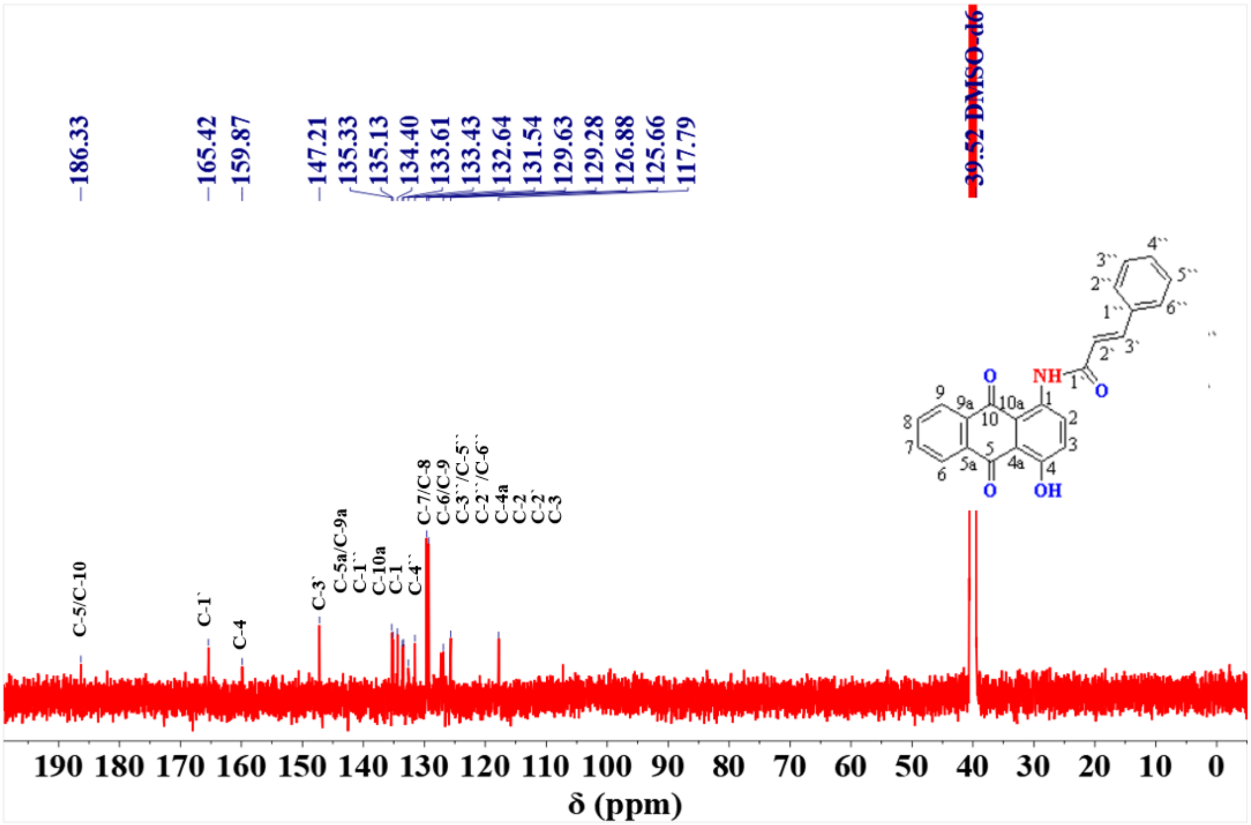

Figure S5B. The ^{13}C NMR spectrum of compound M40. NMR instrument (500 MHz), solvent used is (DMSO-d6).

Figures S6A-C. The ^{1}H NMR, ^{13}C NMR, and DEPT ^{13}C-NMR spectra of M41.

- ***N*-(5-Hydroxy-naphthalen-1-yl)-3-phenyl-acrylamide** (**M41**)

Faint yellow powder; yield (60 %); Mp 145-147 °C, mobile phase (Column chromatography) (EtOAc: n-hexane) (20:80); Rf =0.40; **^{1}H-NMR (500 MHz, DMSO-d$_6$)** δ (ppm): 3.3 (s, 1H, OH), 6.9 (d, J = 7.0 Hz, 1H, Ar-H2), 7.0 (d, J = 16.1 Hz, 1H, H2\`), 7.3 (d, $J$ = 7.3 Hz, 1H, Ar-H8), 7.3 – 7.5 (m, Ar-5H, H6+H7+H3+H2\`\`/H6\`\`), 7.8 – 7.9 (m, 3H, Ar-H3\`\`/H5\`\`+H4\`\`), 7.9 (d, J = 16.1

Hz, 1H, H3`), 8.3 (d, *J* = 8.4 Hz, 1H, Ar-H4).**$^{13}$C-NMR (125 MHz, DMSO-$d_6$)** $\delta$ (ppm): 112.9 (C6), 114.1 (C2), 117.0 (C8), 118.3 (C4), 122.2 (C2`), 123.9 (C3), 127.2 (C1), 127.3 (C2``/C6``), 128.7 (C3``/C5``), 129.0 (C4``), 130.5 (C8a), 130.9 (C4a), 133.9 (C1``), 146.1 (C3`), 146.6 (C5), 153.4 (C7), 165.2 (C1`).

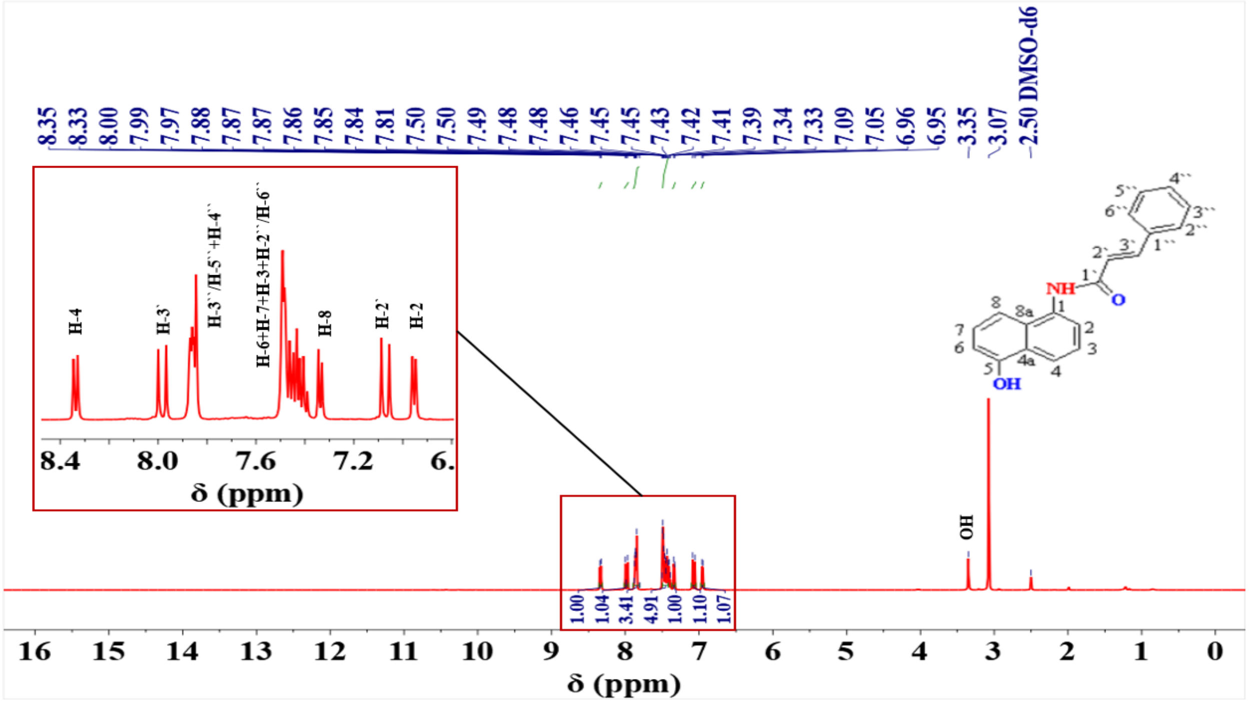

Figure S6A. The ^{1}H NMR spectrum of compound M41. ^{1}H NMR instrument (500 MHz), solvent used is (DMSO-d6).

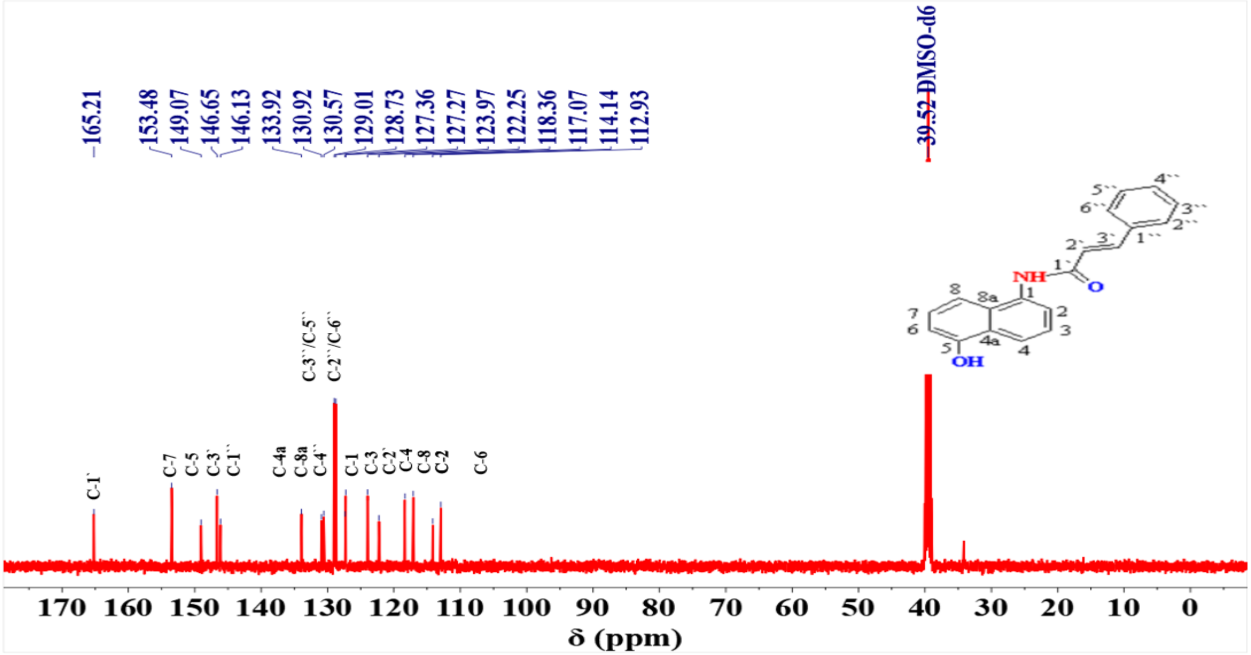

Figure S6B. The ^{13}C NMR spectrum of compound M41. NMR instrument (500 MHz), solvent used is (DMSO-d6).

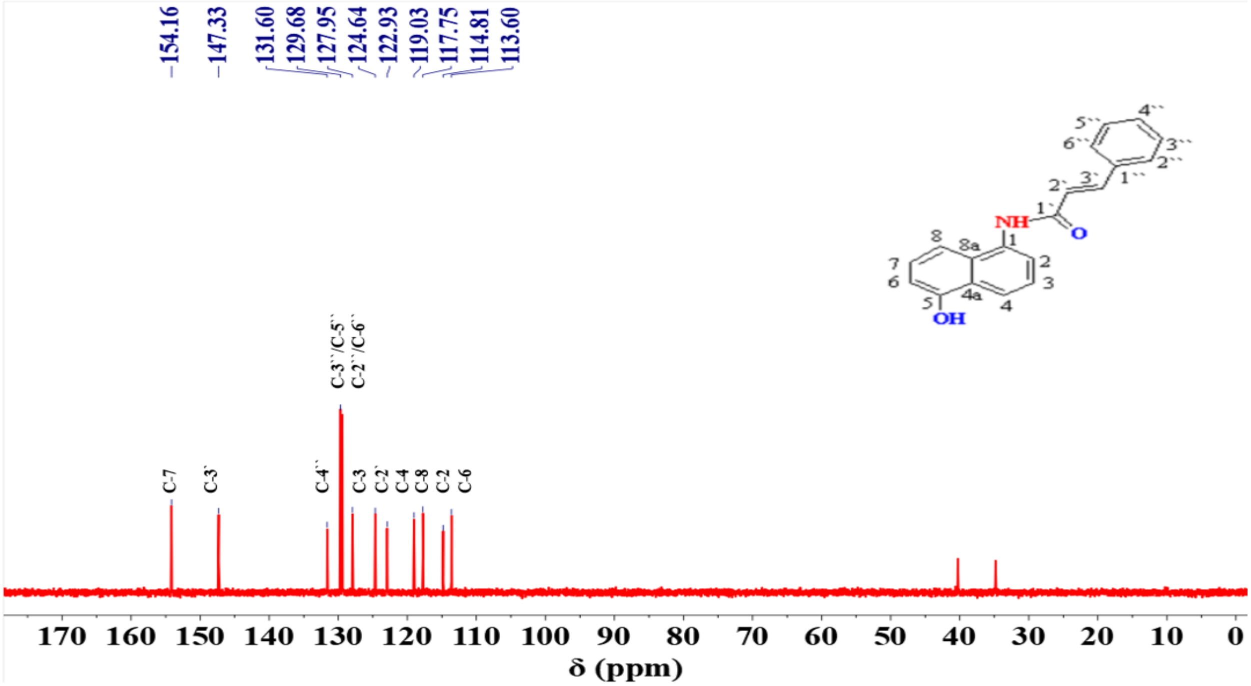

Figure S6C. The DEPT ^{13}C-NMR spectrum of compound M41.

Incubation Time	M34 (IC_{50})	M34 (1/2 IC_{50})	M34 (1/4 IC_{50})	A549 Control
0 h	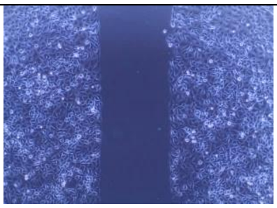	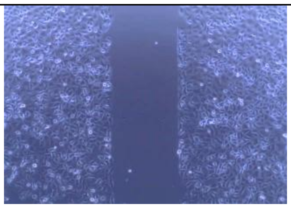	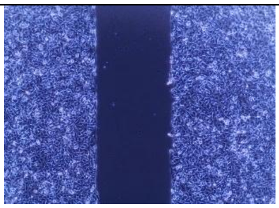	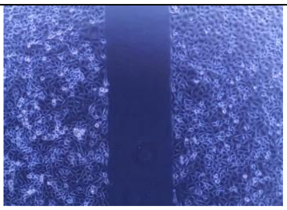
24 h	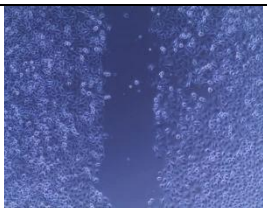	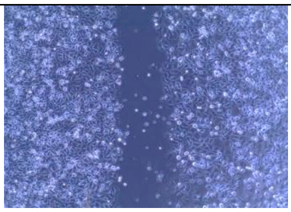	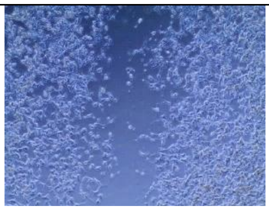	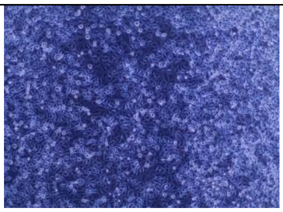
	M8 (IC_{50})	M8 (1/2 IC_{50})	M8 (1/4 IC_{50})	A549 Control
0 H	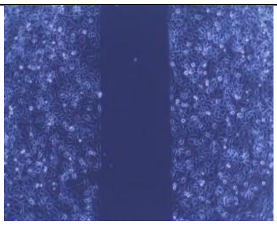	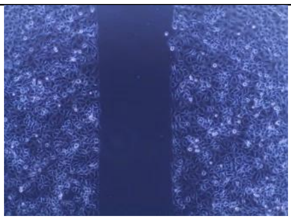	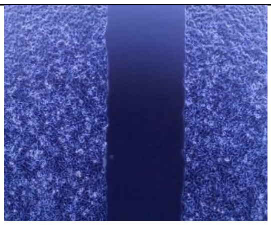	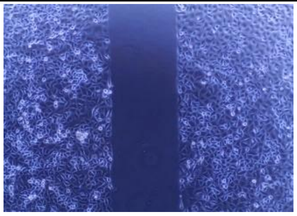
24 H	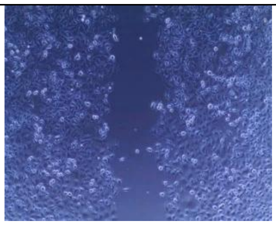	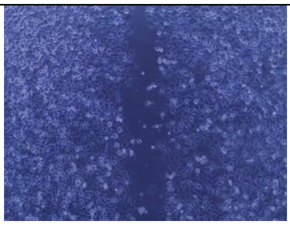	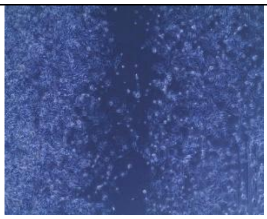	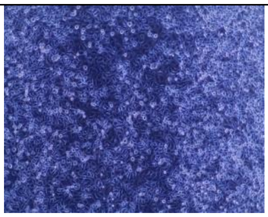
	M9 (IC_{50})	M9 (1/2 IC_{50})	M9 (1/2 IC_{50})	A549 Control
0 H	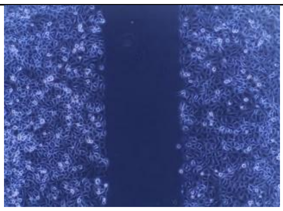	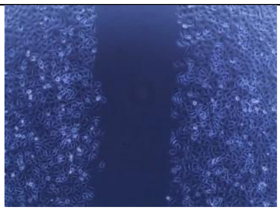	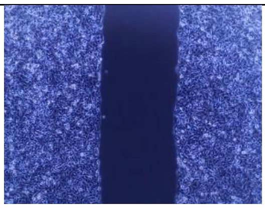	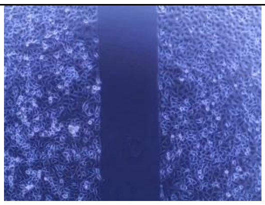

24 H	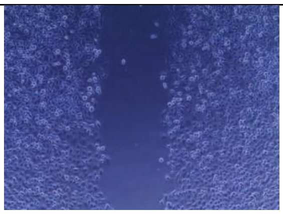	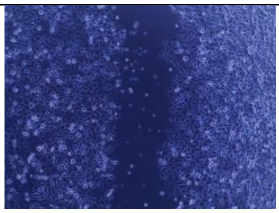	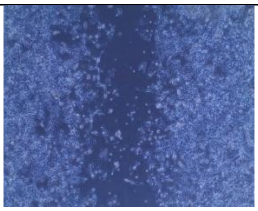	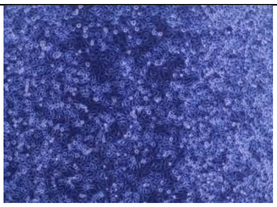
	M10 (IC$_{50}$)	M10 (1/2 IC$_{50}$)	M10 (1/2 IC$_{50}$)	A549 Control
0 H	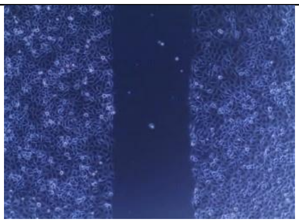	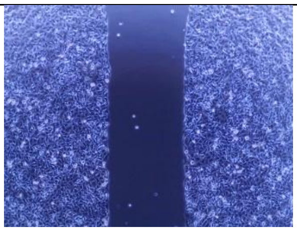	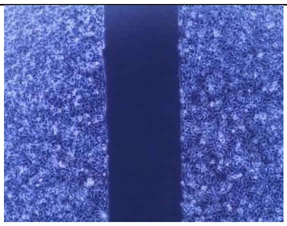	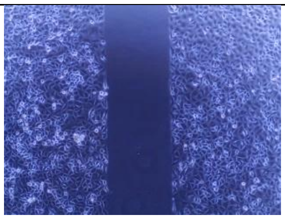
24 H	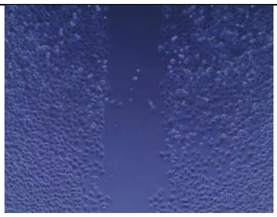	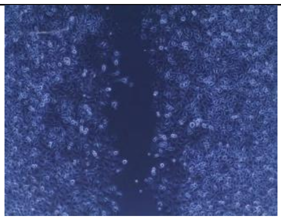	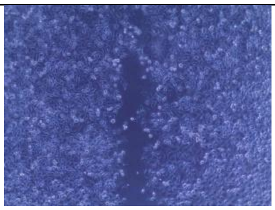	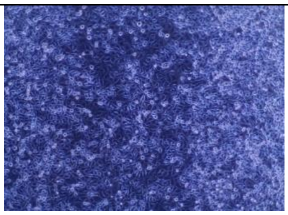
	M27 (IC$_{50}$)	M27 (1/2 IC$_{50}$)	M27 (1/4 IC$_{50}$)	A549 Control
0 H	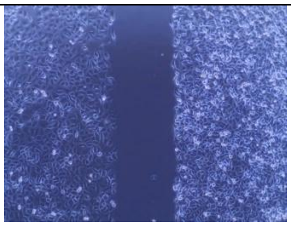	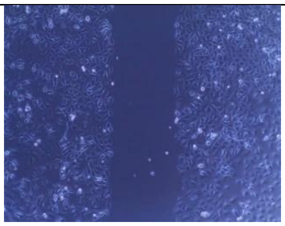	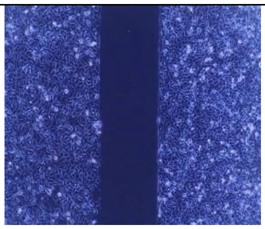	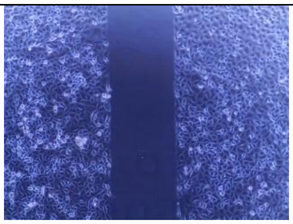
24 H	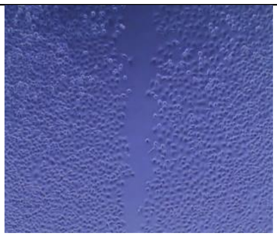	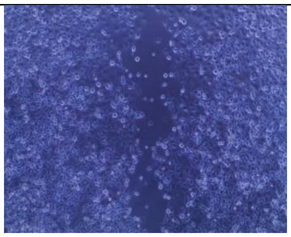	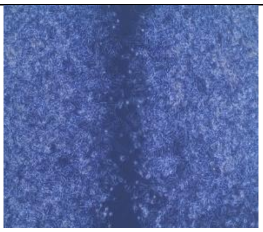	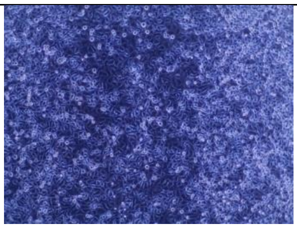
	M1 (IC$_{50}$)	M1 (1/2 IC$_{50}$)	M1 (1/4 IC$_{50}$)	A549 Control

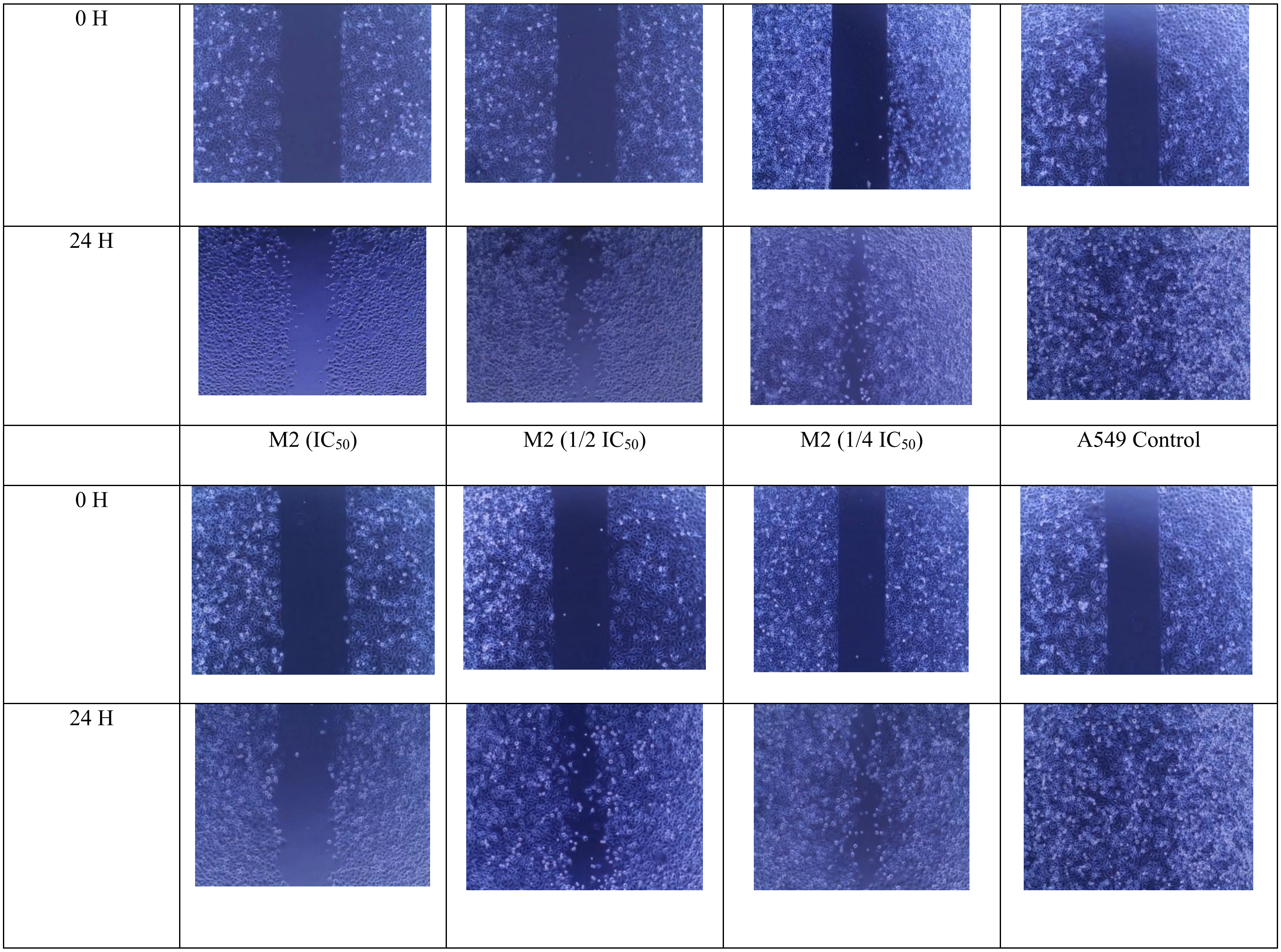

Figure S7. The effect of MMP9 inhibitors on A549 cell migration.

	M34 (IC_{50})	M34 (1/2 IC_{50})	M34 (1/4 IC_{50})	A549 Control
4X	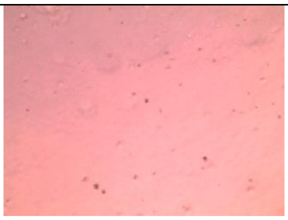	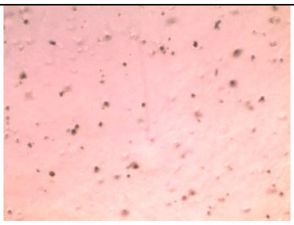	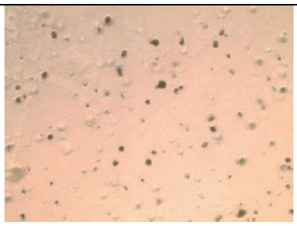	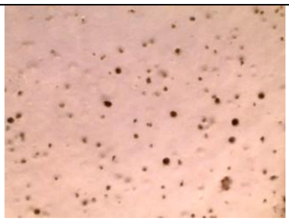
10X	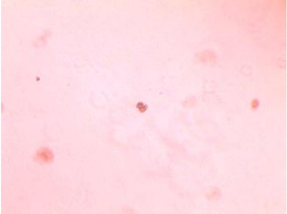	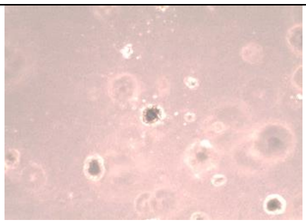	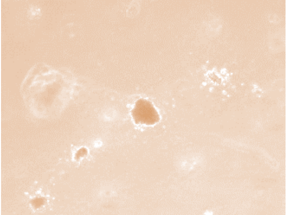	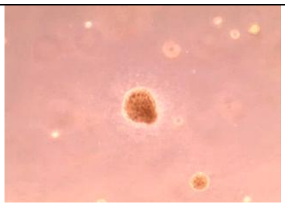
20X	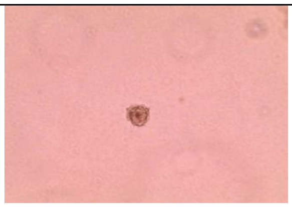	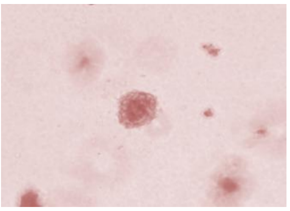	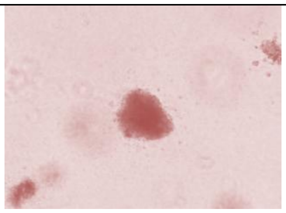	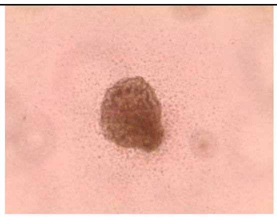
	M8 (IC_{50})	M8 (1/2 IC_{50})	M8 (1/4 IC_{50})	A549 Control
4X	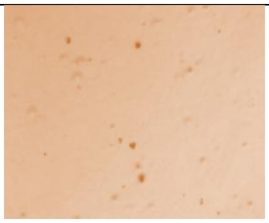	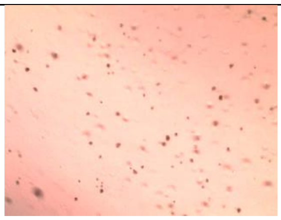	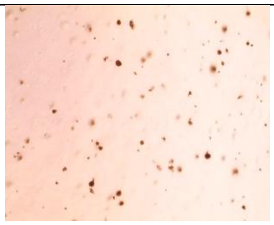	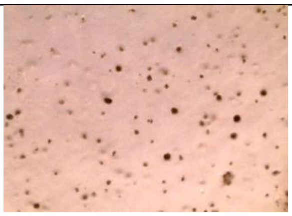
10X	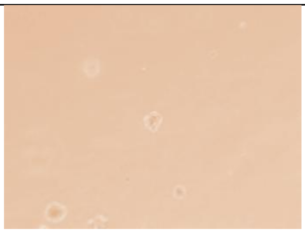	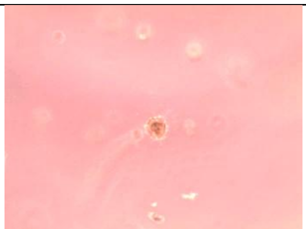	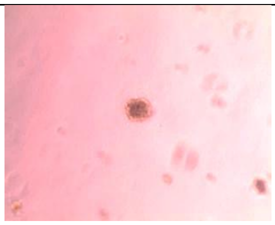	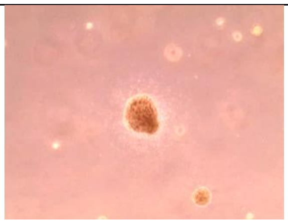
20X	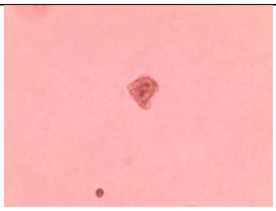	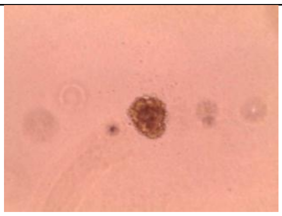	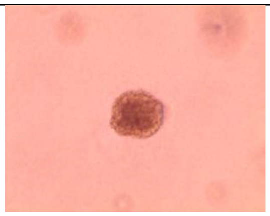	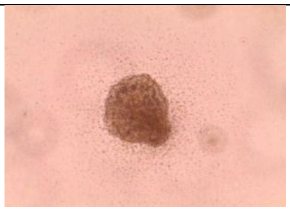
	M9 (IC_{50})	M9 (1/2 IC_{50})	M9 (1/4 IC_{50})	A549 Control

4X	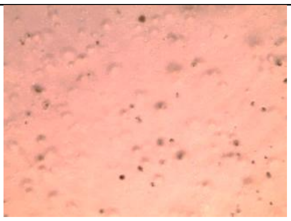	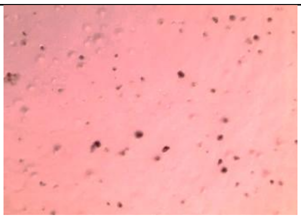	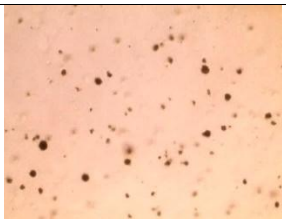	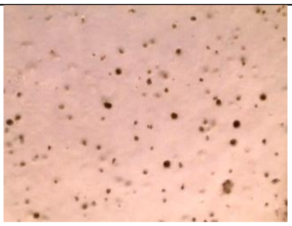
10X	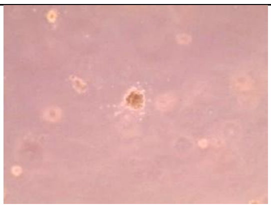	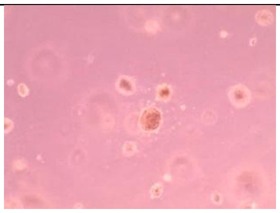	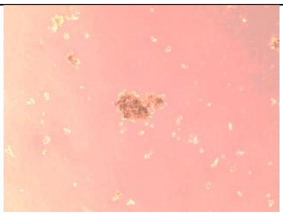	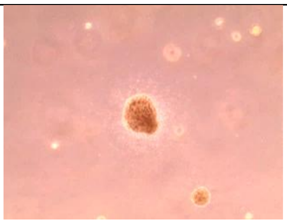
20X	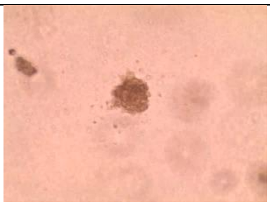	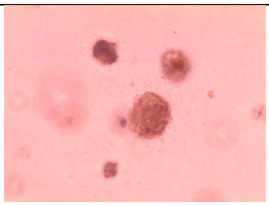	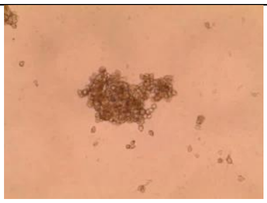	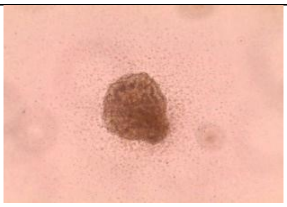
	M10 (IC_{50})	M10 (1/2 IC_{50})	M10 (1/4 IC_{50})	A549 Control
4X	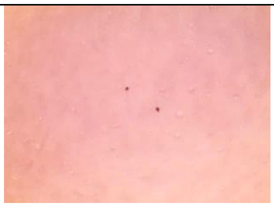	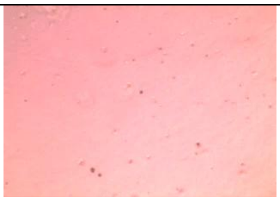	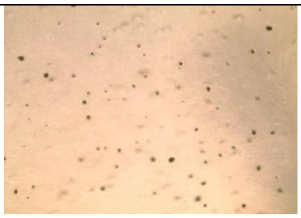	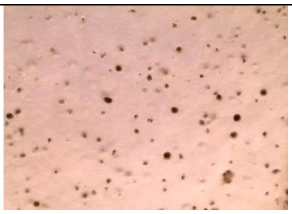
10X	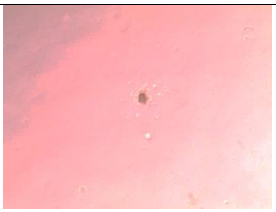	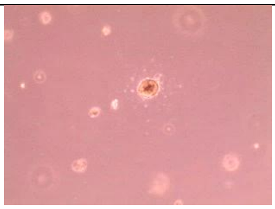	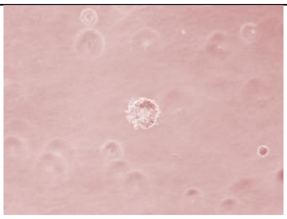	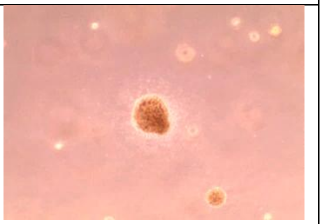
20X	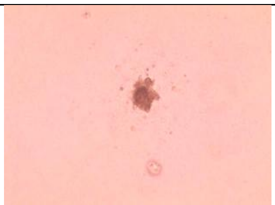	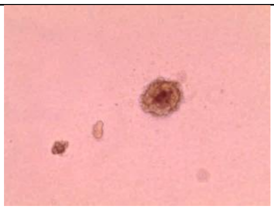	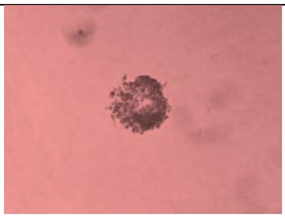	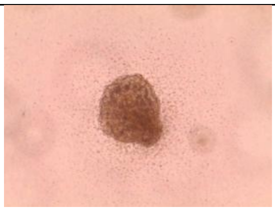
	M27 (IC_{50})	M27 (1/2 IC_{50})	M27 (1/4 IC_{50})	A549 Control

4X	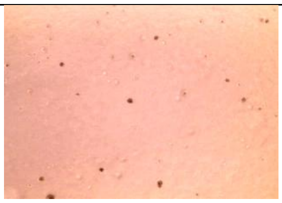	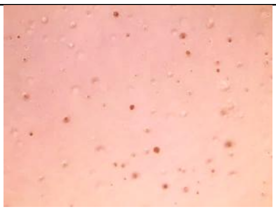	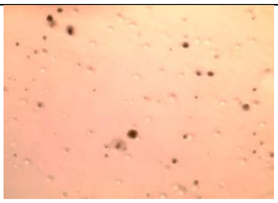	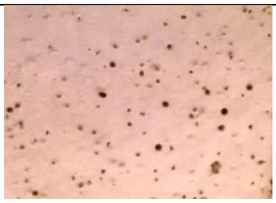
10X	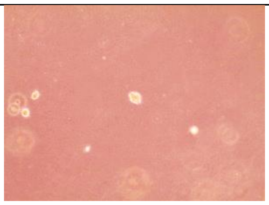	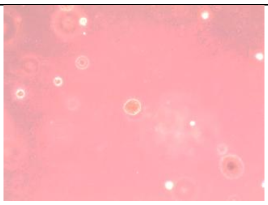	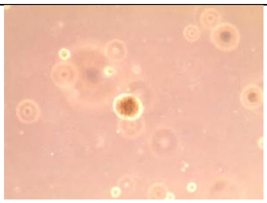	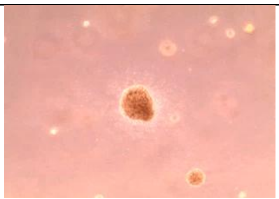
20X	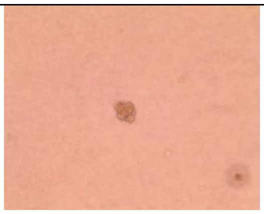	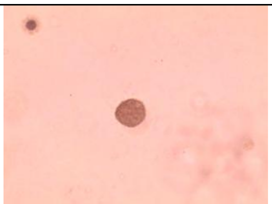	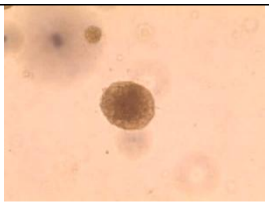	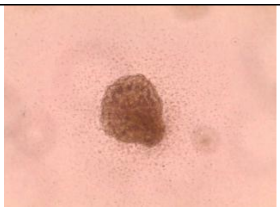
	M1 (IC_{50})	M1 (1/2 IC_{50})	M1 (1/4 IC_{50})	A549 Control
4X	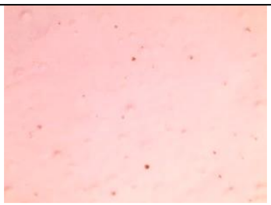	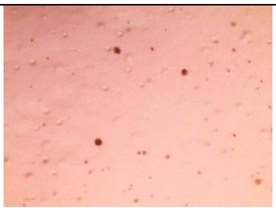	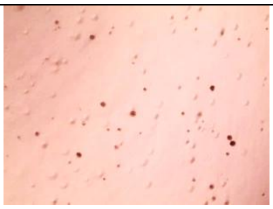	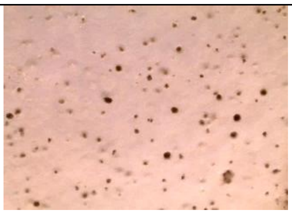
10X	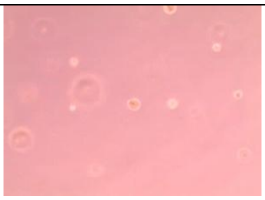	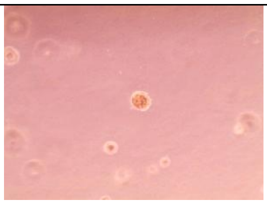	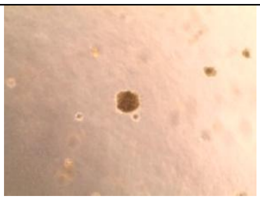	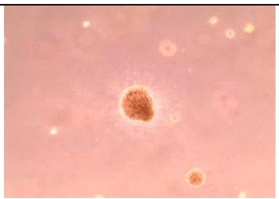
20X	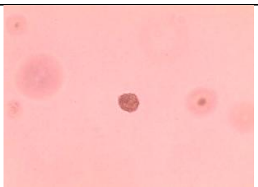	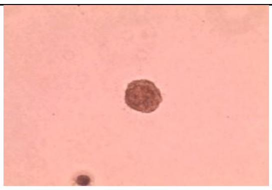	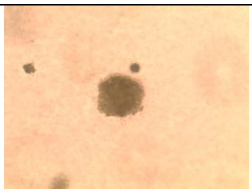	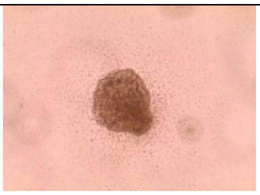
	M2 (IC_{50})	M2 (1/2 IC_{50})	M2 (1/4 IC_{50})	A549 Control

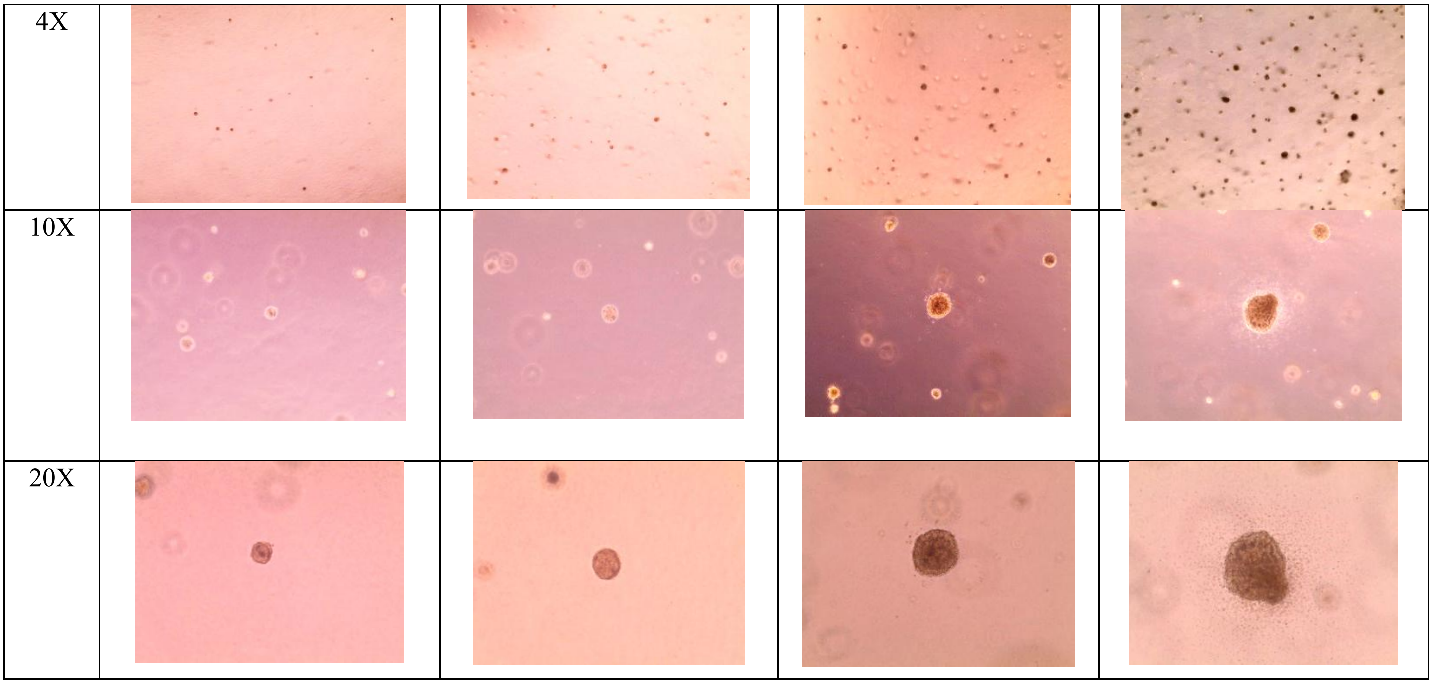

Figure S8. Effect of MMP9 inhibitors on the anchorage-independent growth of A549 lung cancer cell line using colony formation assay. Images show colonies of A549 at different magnifications.

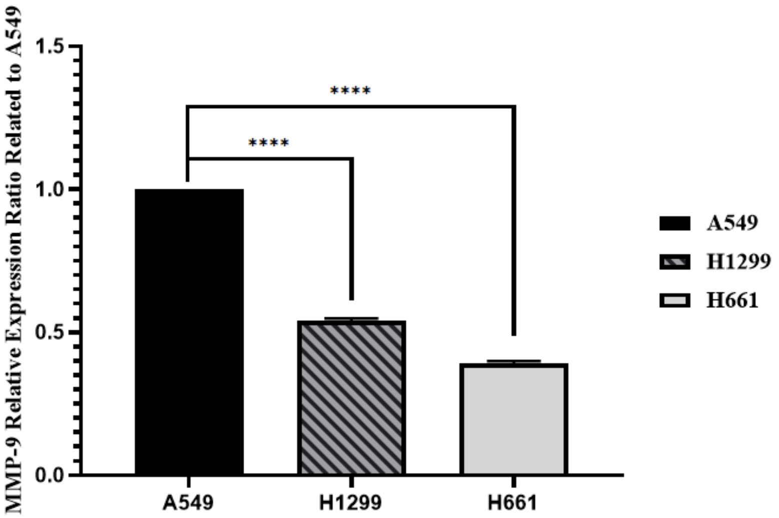

Figure S9. Relative *MMP9* gene expression variation between A549, H1299, and H66 lung cancer cell lines is related to A549 lung cancer cells. Fold difference expressed as mean±SD and was measured using ΔΔCt method. *MMP9*: matrix metalloproteinase-9.

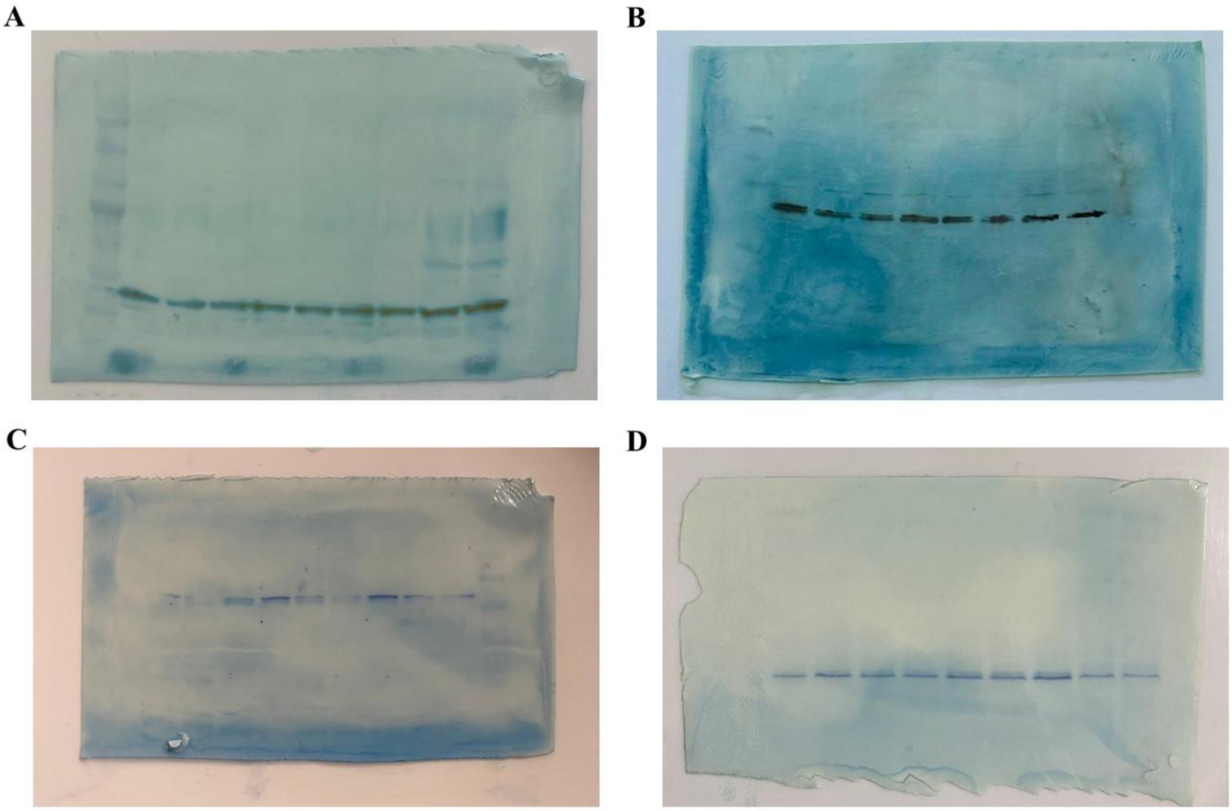

Figure S10. Full-length, uncropped Western blots corresponding to Figure 4C.

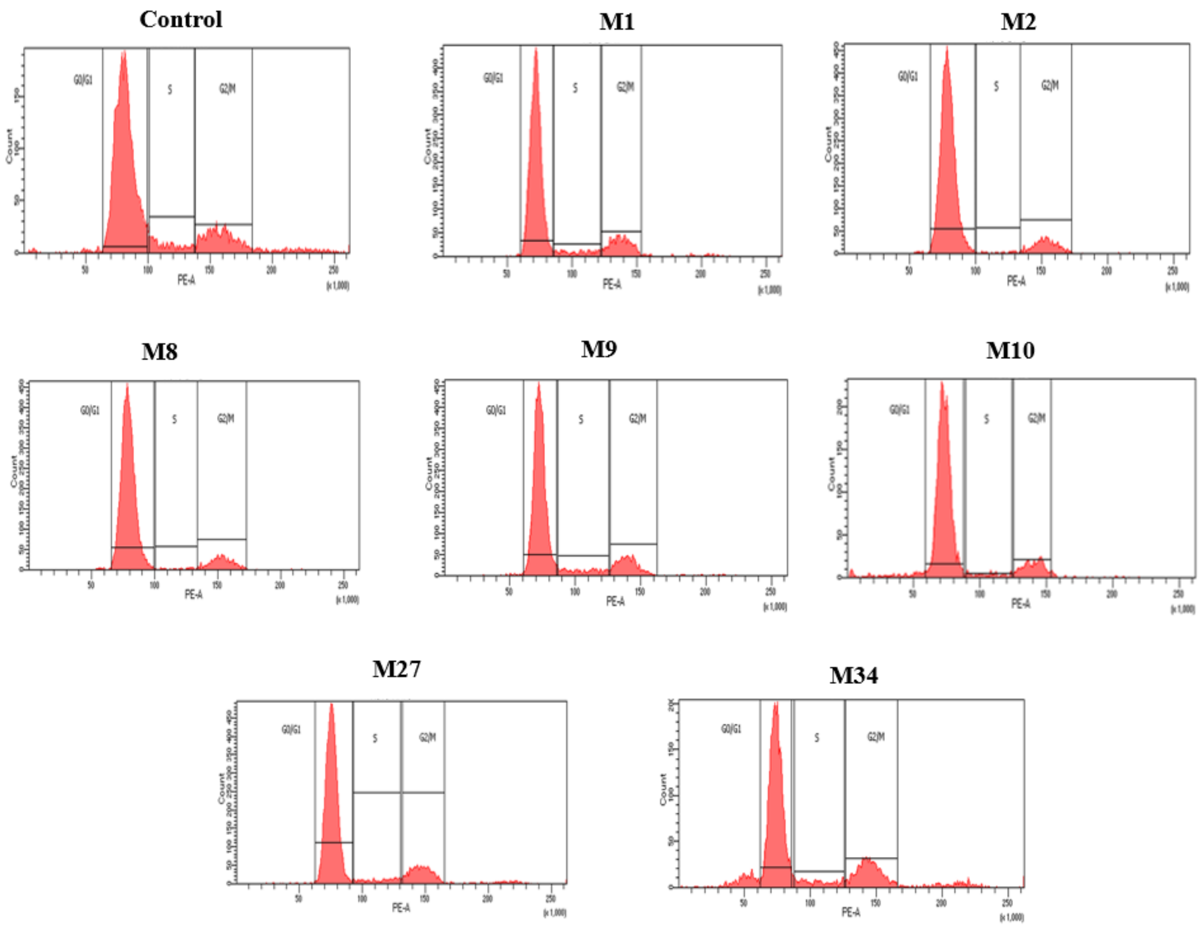

Figure S11. Effect of MMP9 inhibitors for 48 hours using ½ IC_{50} treatment on the cell cycle of the A549 cell line. Histogram of DNA content upon PI staining of respective samples showing G0/G1, S, and G2 phases of the cell cycle. µM: micromolar.

<table>
<tr><td>M1</td><td>M2</td></tr>
<tr><td>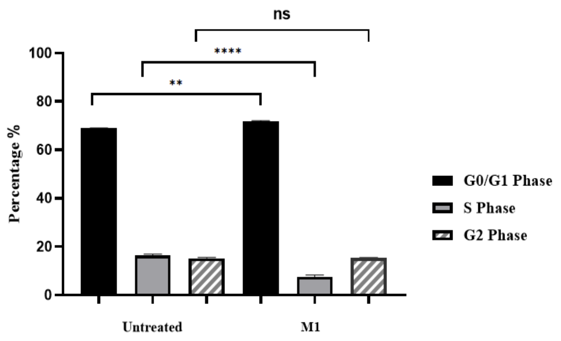</td><td>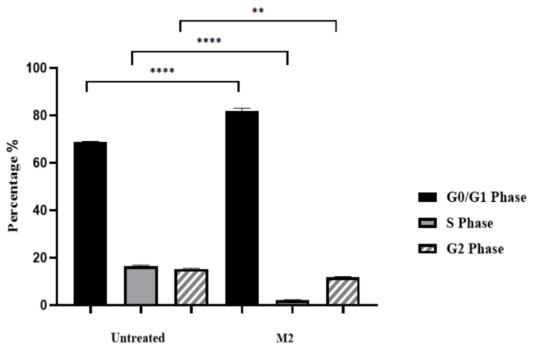</td></tr>
<tr><td>M8</td><td>M9</td></tr>
<tr><td>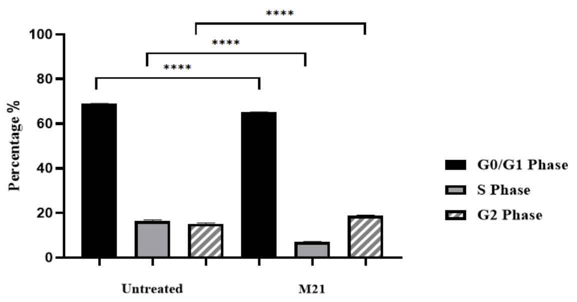</td><td>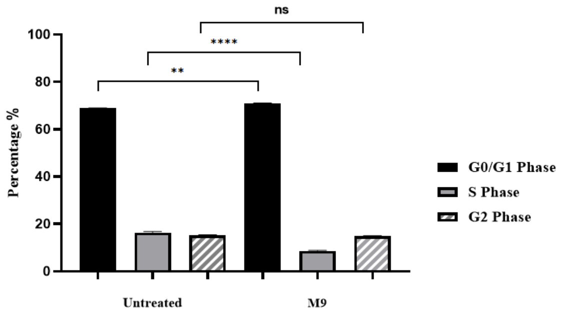</td></tr>
<tr><td>M10</td><td>M27</td></tr>
<tr><td>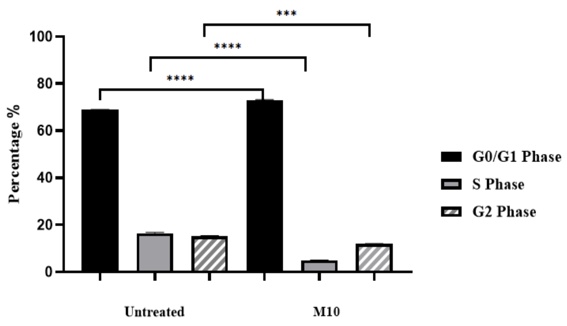</td><td>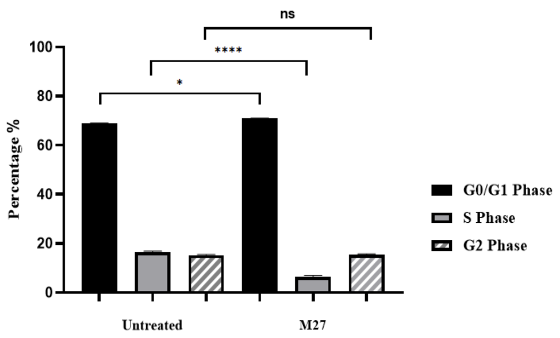</td></tr>
<tr><td colspan="2">M34</td></tr>
</table>

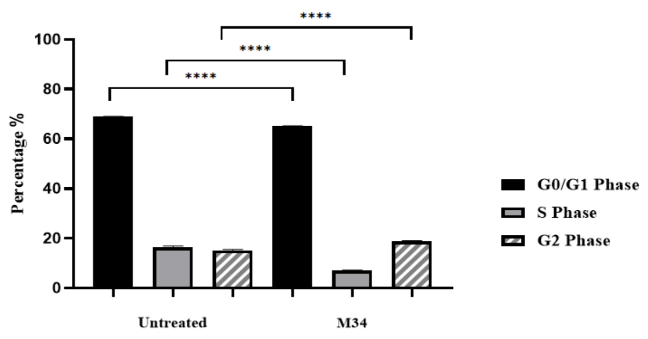

Figure S12. Percentages of cell cycle phases, effect of MMP9 inhibitors for 48 hours using ½ IC_{50} treatment on the cell cycle of lung cancer cell line A549. Percentages represent DNA content upon PI staining of respective samples showing G0/G1, S, and G2 of the cell cycle. *P*-value < 0.05 expresses significantly different from respective untreated cells' status; while asterisk: ns (not significant) P > 0.05; * P ≤ 0.05; ** P ≤ 0.01; *** P ≤ 0.001; **** P ≤ 0.0001 (according to GraphPad Prism 9).

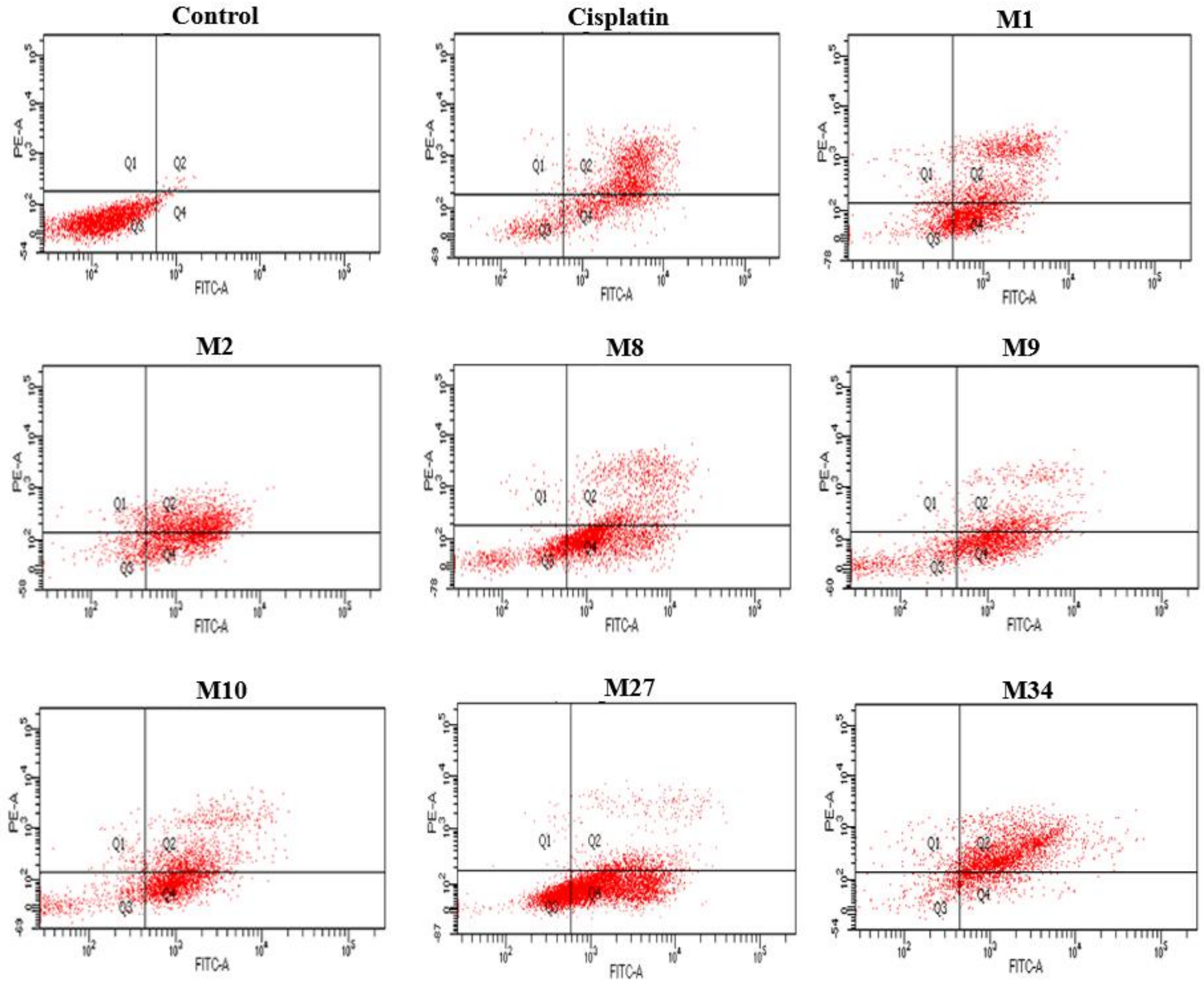

Figure S13. Apoptosis analysis by Annexin V-FITC/PI dual staining in A549 cells. Representative dot plots show the distribution of cells across four populations: viable (Annexin^{-}/PI^{-}, lower left), early apoptotic (Annexin^{+}/PI^{-}, lower right), late apoptotic (Annexin^{+}/PI^{+}, upper right), and necrotic (Annexin^{-}/PI^{+}, upper left).

M1	M2
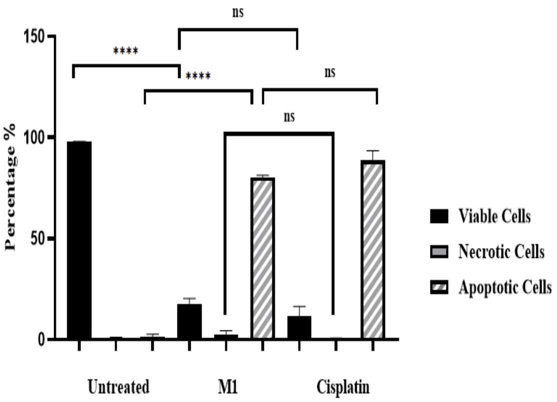	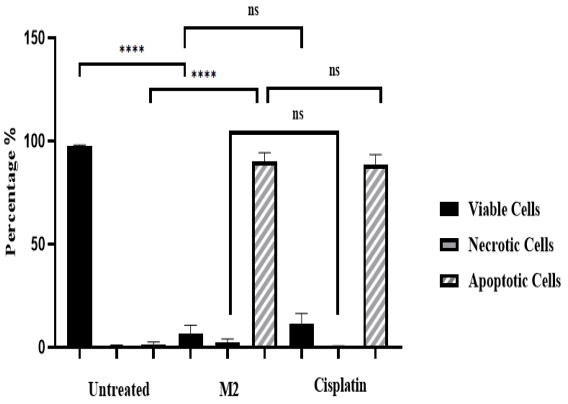

M8	M9
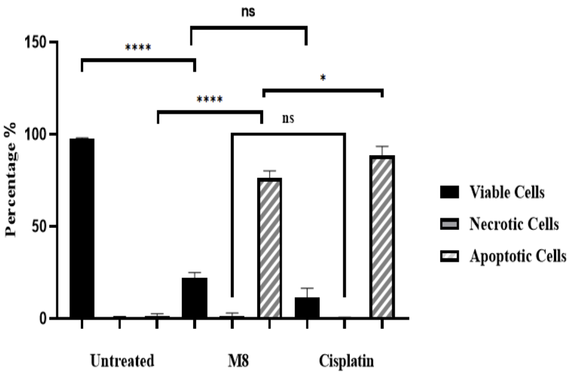	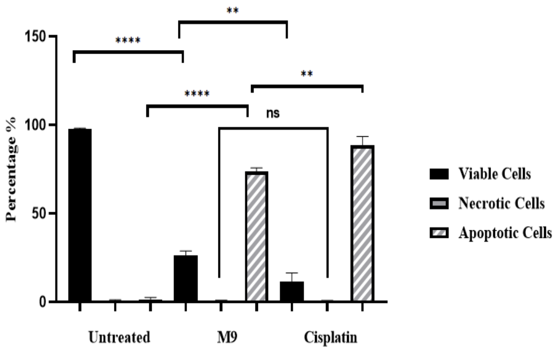

M10	M27
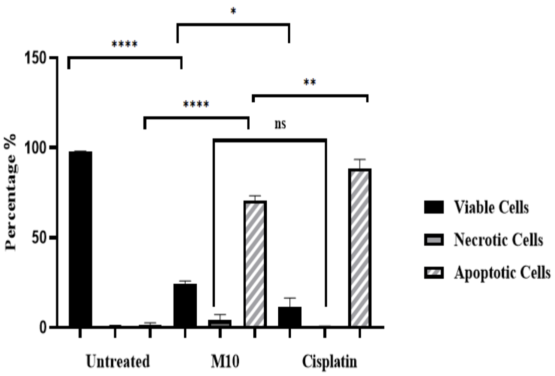	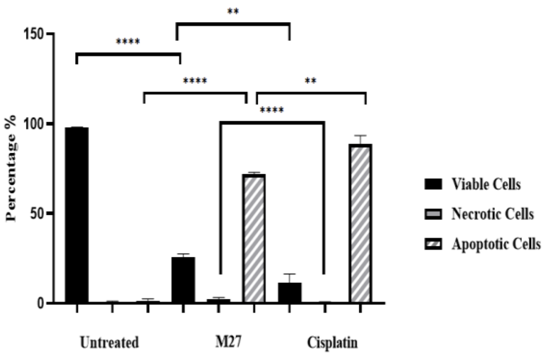

M34

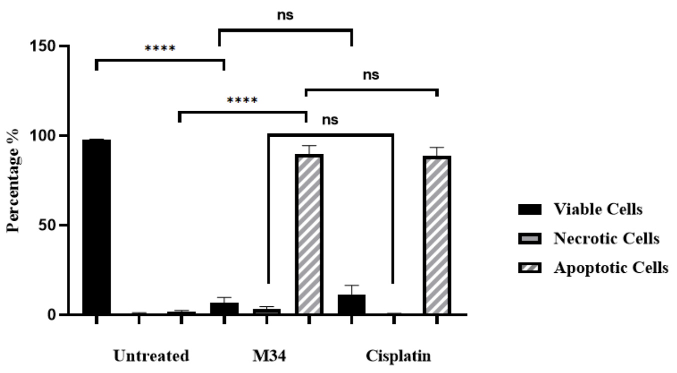

Figure S14. Percentages of healthy, apoptotic, and necrotic cells expressed as mean ± SD, expressing the effect of MMP9 inhibitor (double IC50) treatment on apoptosis of lung cancer cell lines, A549.The experiment was performed in two independent trials. *P*-value < 0.05 expresses significantly different from respective untreated cells' status; while asterisk: ns (not-significant) P > 0.05; * P ≤ 0.05; ** P ≤ 0.01; *** P ≤ 0.001; **** P ≤ 0.0001 (according to GraphPad Prism 9).

Table S1. The primer`s sequence and its optimized annealing temperature (Ta).

No.	Primer	Primer Sequence	Ta (ºC)
1.	MMP9	Forward: 5-GCCACTACTGTGCCTTTGA GTC-3<br>Reverse: 5-CCCTCAGAGAATCGCCAGTACT-3	58.5
2.	VEGF-A	Forward: 5- CTCACCAAGGCCAGCACATAGG-3<br>Reverse: 5-ATCTGGTTCCGAAAACCCTGAG-3	58.5
3.	E-cadherin	Forward: 5-CAGAAAGTTTTCCACCAAAG-3<br>Reverse: 5-ACTGAACCTGACCGTACAAAAT	58.5
4.	CCL2	Forward: 5-ATG AAA GTC TCT GCC GCC CTT CTG T-3<br>Reverse: 5-AGT CTT CGG AGT TTG GGT TTG CTT G-3	58.5
5.	COL6A2	Forward: 5-AACGGATCGGCCCTCTGTG-3<br>Reverse: 5-GCCAGAGCTGTGTTATCCCAA-3	58.5
6.	APP	Forward: 5-AACCAGTGACCATCCAGAAC-3<br>Reverse: 5-ACTTGTCAGGAACGAGAAGG-3	58.5
7.	CCL17	Forward: 5-TCC CCT TAG AAA GCT GAA GAC-3<br>Reverse: 5- ACT GCA TTC TTC ACT CTC TTG-3	58.5
8.	ETV4	Forward: 5-CAGCTCAGCTTCTTCCTAGGT-3<br>Reverse: 5-CCTCTCTGCTTATACCCAGCAC-3.	58.5
9.	COL5A1	Forward: 5-CTACATCCGTGCCCTGGT-3<br>Reverse: 5-CCAGCACCGTCTTCTGGTAG-3	58.5

Ta: annealing temperature; MMP9: matrix metalloproteinase-9; VEGF-A: gene for vascular endothelial growth factor A; E-cadherin: gene for epithelial cadherin; CCL2: gene for chemokine (C-C motif) ligand 2; COL6A2: gene for Collagen alpha-2(VI); APP: gene for Amyloid-beta precursor protein; CCL17: gene for C-C Motif Chemokine Ligand 17; ETV4: gene for polyoma enhancer activator 3; COL5A1: gene for Collagen Type V Alpha 1 Chain.

Table S2. Gene overlap among the seven MMP9-related lung cancer pathway maps.

Overlapping Pathway Maps	No. Overlapping Pathways	No. Overlapping Network Objects	Overlapping Network Objects	Gene Symbols
Map1, Map2, Map3, Map4 Map5, Map6, Map7	7	1	MMP9	*MMP9*
Map1, Map2, Map5, Map7	4	1	VEGF-A	*VEGFA*
Map1, Map4, Map5, Map7	4	1	COX-2	*PTGS2*
Map2, Map3, Map5, Map7	4	1	MMP-2	*MMP2*
Map3, Map4, Map5, Map6	4	1	MEKK1	*MAP3K1*
Map1, Map2, Map4	3	1	mTOR	*MTOR*
Map1, Map2, Map5	3	2	PI3K, p70	*PIK3CA, RPS6KB1*
Map1, Map3, Map5	3	2	c-Src, c-Fos	*SRC, FOS*
Map1, Map4, Map5	3	1	PI3K	*PIK3CA*
Map2, Map3, Map4	3	3	MEK1, MEK2, c-Raf-1	*MAP2K1, MAP2K2*
Map2, Map3, Map5	3	2	H-Ras, CREB1	*HRAS, CREB1*
Map3, Map5, Map7	3	1	p38	*MAPK14*
Map4, Map5, Map6	3	2	Beta-catenin, Rac1	*CTNNB1, RAC1*
Map4, Map6, Map7	3	1	E-cadherin	*CDH1*
Map1, Map2	2	1	Bcl-XL	*BCL2L1*
Map1, Map3	2	2	EGF, ADAM17	*EGF, ADAM17*
Map1, Map4	2	2	microRNA 21, PTEN	*MIR21, PTEN*
Map1, Map5	2	6	HGF receptor, STAT3, HIF1A, HGF, IL-6, SP1	*MET, STAT3, HIF1A, HGF, IL6, SP1*
Map1, Map7	2	2	IKK-alpha, EGR1	*CHUK, EGR1*
Map2, Map5	2	1	p90Rsk	*RPS6KA1*
Map2, Map6	2	2	ERK5, MAP2K5	*MAPK7, MAP2K5*

Map3, Map4	2	2	MEK4, JNK	*MAP2K4, MAPK8-10*
Map3, Map5	2	1	MEK6	*MAP2K6*
Map4, Map5	2	2	2K-RAS, Tiam1	*KRAS*
Map4, Map7	2	1	PI3K	*TIAM1*
Map5, Map6	2	1	GSK3 beta	*GSK3B*
Map5, Map7	2	1	IL-1 beta	*IL1B*
Map6, Map7	2	3	E-cadherin, Vimentin, MMP9	*CDH1, VIM, MMP9*

The analyzed pathway maps include: EGFR Signaling Pathway in Lung Cancer (Map1), IGF Signaling in Lung Cancer (Map2), Influence of Smoking on Activation of EGFR Signaling in Lung Cancer Cells (Map3), K-RAS Signaling in Lung Cancer (Map4), Regulation and Signaling of HGF Receptor (Met) and MSP Receptor (RON) in Lung Cancer (Map5), Role of inhibition of WNT signaling in the progression of lung cancer (Map6), and Stimulation of TGF-β signaling in lung cancer (Map7). Gene symbols follow HGNC convention.

Table S3. The IC_{50} values for MMP9 inhibitors in A549, H1299, and H661 lung cancer cell lines at 48, 72, and 96 h. Experiments were performed in duplicate three times independently (n=6). h: hour; µM: micromolar.

IC_{50} (µM)										
Compound		A549			H1299			H661		
No.	Code	48 h	72 h	96 h	48 h	72 h	96 h	48 h	72 h	96 h
1.	M1	1189 ± 4	110 ± 7	83 ± 4	671 ± 6	163 ± 6	85 ± 4	162 ± 6	110 ± 7	75 ± 4
2.	M2	298 ± 6	40 ± 5	28 ± 8	99 ± 4	83 ± 7	59 ± 3	91 ± 4	63 ± 3	67 ± 8
3.	M5	1557 ± 9	390 ± 4	218 ± 6	231 ± 6	186 ± 4	524 ± 5	197 ± 6	119 ± 4	177 ± 2
4.	M6	2233 ± 6	349 ± 8	100 ± 4	1283 ± 5	374 ± 4	329 ± 3	4773 ± 4	102 ± 8	724 ± 5
5.	M7	336 ± 6	228.9 ± 2	140.7 ± 4	774.2 ± 6	254.9 ± 4	200 ± 8	647 ± 2	347 ± 4	362 ± 3
6.	M8	175 ± 4	102 ± 6	44.98 ± 8	346.9 ± 4	190 ± 3	167 ± 2	225 ± 4	140 ± 5	94 ± 2
7.	M9	200 ± 9	115 ± 6	61.22 ± 7	234.6 ± 8	190 ± 4	169 ± 8	272 ± 6	236 ± 3	175 ± 4
8.	M10	460 ± 4	96 ± 2	50 ± 9	680 ± 6	233.4 ± 5	160 ± 2	230 ± 4	150 ± 6	80 ± 5

9.	M11	887.7 ± 4	285.5 ± 5	197.8 ± 4	490.8 ± 7	289.4 ± 4	210 ± 5	597 ± 3	271.7 ± 2	111 ± 6
10.	M12	650 ± 6	375 ± 9	366 ± 3	605 ± 4	392 ± 6	281 ± 6	329 ± 5	161 ± 8	331 ± 6
11.	M13	351 ± 4	331 ± 3	308 ± 4	433 ± 2	306 ± 5	331 ± 4	532 ± 4	187 ± 8	132 ± 4
12.	M14	542 ± 6	379 ± 5	65 ± 4	1235 ± 5	276 ± 2	235 ± 8	938 ± 6	175 ± 4	243 ± 6
13.	M15	619 ± 3	311 ± 4	209 ± 6	447 ± 6	505 ± 9	1549 ± 8	931 ± 4	430 ± 9	562 ± 2
14.	M20	10133 ± 2	1369 ± 4	619 ± 5	406 ± 2	326 ± 3	338 ± 5	2458 ± 6	369 ± 5	240 ± 4
15.	M21	301 ± 3	1234 ± 2	522 ± 6	384 ± 6	246 ± 4	333 ± 3	1146 ± 4	363 ± 2	254 ± 3
16.	M22	833 ± 4	1479 ± 5	879 ± 4	1023 ± 5	352 ± 3	265 ± 6	3202 ± 5	353 ± 3	286 ± 4
17.	M23	955 ± 3	221 ± 4	293 ± 6	1206 ± 3	398 ± 6	321 ± 5	1148 ± 8	305 ± 3	278 ± 2
18.	M24	1380 ± 4	836 ± 5	818 ± 3	623 ± 4	503 ± 6	374 ± 3	658 ± 4	483 ± 3	317 ± 6
19.	M25	683 ± 6	118 ± 3	79 ± 4	483 ± 6	422 ± 3	341 ± 4	2098 ± 6	414 ± 3	310 ± 4
20.	M26	614 ± 3	426 ± 4	254 ± 6	1112 ± 4	670 ± 3	576 ± 4	1709 ± 4	338 ± 3	350 ± 4
21.	M27	479 ± 4	100 ± 8	60 ± 3	542 ± 4	655 ± 3	487 ± 8	1683 ± 3	446 ± 4	300 ± 3

22.	M28	310 ± 3	246 ± 8	347 ± 4	277 ± 3	183 ± 8	422 ± 4	2298 ± 8	375 ± 7	333 ± 4
23.	M29	341 ± 4	404 ± 3	89 ± 2	153 ± 8	141 ± 6	197 ± 3	168 ± 4	434 ± 2	105 ± 4
24.	M33	1139 ± 4	1200 ± 8	455 ± 4	350 ± 6	524 ± 9	714 ± 4	777 ± 8	275 ± 2	259 ± 6
25.	M34	139 ± 6	78 ± 7	54 ± 3	147 ± 5	125 ± 2	75 ± 4	177 ± 3	163 ± 4	64 ± 5
26.	M35	2943 ± 4	215 ± 6	177 ± 2	473 ± 4	171 ± 4	390 ± 3	322.8 ± 2	125 ± 3	175 ± 5
27.	M39	19193 ± 6	258 ± 4	247 ± 6	610 ± 3	214 ± 6	394 ± 6	663 ± 2	1399 ± 9	297 ± 5
28.	M40	261 ± 6	226 ± 3	204 ± 2	144 ± 4	91 ± 5	89 ± 2	305 ± 4	115 ± 2	255 ± 6
29.	M41	2943 ± 8	303 ± 6	419 ± 3	144 ± 5	251 ± 4	279 ± 3	119 ± 5	55 ± 4	204 ± 3

Table S4. Quantitative analysis of the effect of MMP9 inhibitors on A549 cell migration. The wound area was measured using ImageJ software. *P*-value < 0.05 expresses significantly different from the respective untreated condition, while asterisk: ns (not-significant) $p > 0.05$; * $P \leq 0.05$; ** $P \leq 0.01$, *** $P \leq 0.001$, **** $P \leq 0.0001$ (according to GraphPad prism 9).

Compound	Concentration (µM)	Wound Closure %	Migration Inhibition %	*P*-Value	Significance
Untreated A549 cells	-	99.9	0.1	-	-
M1	27.5	89.4	11	<0.0001 (****)	Significant
	55	64	36	<0.0001 (****)	Significant
	110	29	71	<0.0001 (****)	Significant
M2	10	85.2	15	<0.0001 (****)	Significant
	20	61.7	38	<0.0001 (****)	Significant
	40	46	54	<0.0001 (****)	Significant
M8	25.5	80.5	20	<0.0001 (****)	Significant
	51	64.5	36	<0.0001 (****)	Significant
	102	34.2	66	<0.0001 (****)	Significant
M9	28.75	55.3	45	<0.0001 (****)	Significant
	57.5	30.6	69	<0.0001 (****)	Significant
	115	12.9	87	<0.0001 (****)	Significant
M10	24	79.6	21	<0.0001 (****)	Significant
	48	47.8	52	<0.0001 (****)	Significant
	96	16.9	83	<0.0001 (****)	Significant
M27	25	87.8	12	<0.0001 (****)	Significant
	50	64.2	36	<0.0001 (****)	Significant
	100	38.7	61	<0.0001 (****)	Significant
M34	19.5	65.5	34.5	<0.0001 (****)	Significant
	39	36.3	64	<0.0001 (****)	Significant
	78	22.86	77	<0.0001 (****)	Significant

Table S5. Effect of MMP9 inhibitors on colony size and count of A549 lung cancer cells using colony formation assay. Cells were exposed to IC_{50}, ½ IC_{50}, or ¼ IC_{50} concentration for 72 h, followed by 14 days of sustained growth in soft agar. Colony size was measured using particle analysis through identifying colony color threshold via ImageJ software (Ver. 1.53e.). *P*-value < 0.05 express significantly different from respective untreated condition; while asterisk: ns (not-significant) P > 0.05; * P ≤ 0.05; ** P ≤ 0.01; *** P ≤ 0.001; **** P ≤ 0.0001 (according to GraphPad Prism 9).

Compound	Concentration (µM)	Colony Count	*P*-Value	Significance	Colony Average Size (Pixels)	*P*-Value	Significance
Control	-	2850	-	-	266261	-	-
M1	¼ IC_{50} (27.5)	1966	<0.0001 (****)	Significant	150223	<0.0001 (****)	Significant
	½ IC_{50} (55)	1145	<0.0001 (****)	Significant	79608	<0.0001 (****)	Significant
	IC_{50} (110)	465	<0.0001 (****)	Significant	27984	<0.0001 (****)	Significant
M2	¼ IC_{50} (10)	1350	<0.0001 (****)	Significant	125877	<0.0001 (****)	Significant
	½ IC_{50} (20)	575	<0.0001 (****)	Significant	44344	<0.0001 (****)	Significant
	IC_{50} (40)	288	<0.0001 (****)	Significant	26966	<0.0001 (****)	Significant
M8	¼ IC_{50} (25.5)	2206	<0.0001 (****)	Significant	199538	<0.0001 (****)	Significant
	½ IC_{50} (51)	1380	<0.0001 (****)	Significant	90264	<0.0001 (****)	Significant
	IC_{50} (102)	470	<0.0001 (****)	Significant	51788	<0.0001 (****)	Significant
M9	¼ IC_{50} (28.75)	2335	<0.0001 (****)	Significant	190582	<0.0001 (****)	Significant
	½ IC_{50} (57.5)	1375	<0.0001 (****)	Significant	118764	<0.0001 (****)	Significant

	IC_{50} (115)	790	<0.0001 (****)	Significant	62136	<0.0001 (****)	Significant
M10	¼ IC_{50} (24)	565	<0.0001 (****)	Significant	175343	<0.0001 (****)	Significant
	½ IC_{50} (48)	250	<0.0001 (****)	Significant	102656	<0.0001 (****)	Significant
	IC_{50} (96)	85	<0.0001 (****)	Significant	48828	<0.0001 (****)	Significant
M27	¼ IC_{50} (25)	2340	<0.0001 (****)	Significant	140772	<0.0001 (****)	Significant
	½ IC_{50} (50)	995	<0.0001 (****)	Significant	60896	<0.0001 (****)	Significant
	IC_{50} (100)	530	<0.0001 (****)	Significant	30884	<0.0001 (****)	Significant
M34	¼ IC_{50} (19.5)	2265	<0.0001 (****)	Significant	197753	<0.0001 (****)	Significant
	½ IC_{50} (39)	1240	<0.0001 (****)	Significant	77877	<0.0001 (****)	Significant
	IC_{50} (78)	340	<0.0001 (****)	Significant	26212	<0.0001 (****)	Significant
